# Targeting lncRNA DDIT4‐AS1 Sensitizes Triple Negative Breast Cancer to Chemotherapy via Suppressing of Autophagy

**DOI:** 10.1002/advs.202207257

**Published:** 2023-04-25

**Authors:** Ting Jiang, Jiaojiao Zhu, Shilong Jiang, Zonglin Chen, Ping Xu, Rong Gong, Changxin Zhong, Yueying Cheng, Xinyuan Sun, Wenjun Yi, Jinming Yang, Wenhu Zhou, Yan Cheng

**Affiliations:** ^1^ Department of Pharmacy The Second Xiangya Hospital Central South University Changsha 410011 China; ^2^ Xiangya School of Pharmaceutical Sciences Central South University Changsha 410008 China; ^3^ Hunan Provincial Engineering Research Centre of Translational Medicine and Innovative Drug Changsha 410011 China; ^4^ Department of Pharmacy Xiangya Hospital Central South University Changsha 410008 China; ^5^ Department of General Surgery The Second Xiangya Hospital Central South University Changsha 410011 China; ^6^ Department of Cancer Biology and Toxicology Department of Pharmacology College of Medicine and Markey Cancer Center University of Kentucky Lexington KY 40536 USA; ^7^ Key Laboratory of Diabetes Immunology (Central South University) Ministry of Education Changsha 410011 China

**Keywords:** autophagy, chemotherapy, lncRNA DDIT4‐AS1, nanoparticle, TNBC

## Abstract

In this study, it is found that the lncRNA, DNA damage inducible transcript 4 antisense RNA1 (DDIT4‐AS1), is highly expressed in triple‐negative breast cancer (TNBC) cell lines and tissues due to H3K27 acetylation in the promoter region, and promotes the proliferation, migration, and invasion of TNBC cells via activating autophagy. Mechanistically, it is shown that DDIT4‐AS1 induces autophagy by stabilizing DDIT4 mRNA via recruiting the RNA binding protein AUF1 and promoting the interaction between DDIT4 mRNA and AUF1, thereby inhibiting mTOR signaling pathway. Furthermore, silencing of DDIT4‐AS1 enhances the sensitivity of TNBC cells to chemotherapeutic agents such as paclitaxel both in vitro and in vivo. Using a self‐activatable siRNA/drug core–shell nanoparticle system, which effectively deliver both DDIT4‐AS1 siRNA and paclitaxel to the tumor‐bearing mice, a significantly enhanced antitumor activity is achieved. Importantly, the codelivery nanoparticles exert a stronger antitumor effect on breast cancer patient‐derived organoids. These findings indicate that lncRNA DDIT4‐AS1‐mediated activation of autophagy promotes progression and chemoresistance of TNBC, and targeting of DDIT4‐AS1 may be exploited as a new therapeutic approach to enhancing the efficacy of chemotherapy against TNBC.

## Graphical abstract

DNA damage inducible transcript 4 antisense RNA1 (DDIT4‐AS1) is identified as a oncogenic lncRNA in triple‐negative breast cancer (TNBC). Mechanistically, DDIT4‐AS1 stabilizes DDIT4 mRNA via recruiting the RNA binding protein AUF1, which subsequently inactivates mTORC1 and induces cyto‐protective autophagy. A smart metal–organic framework nanosystem is fabricated to effectively load DDIT4‐AS1 siRNA and paclitaxel (PTX) for the treatment of TNBC.

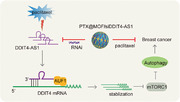

## 1 Introduction

Triple‐negative breast cancer (TNBC), a type of breast cancer devoid of expression of estrogen receptor (ER), progesterone receptor (PR), and human epidermal growth factor receptor‐2 (HER2),^[^ ^1^ ^]^ has a highly aggressive clinical course with earlier age of onset, greater metastatic potential and poorer clinical outcomes.^[^ ^2^ ^]^ Due to its special molecular phenotype, TNBC is impervious to endocrine therapy or molecular targeted therapy. Therefore, chemotherapy such as taxanes, anthracycline, and cisplatin is the main systemic treatment for this disease. Nevertheless, chemoresistance and low response rates (10–15%) often hamper success of the treatment.^[^ ^1^ ^]^

Autophagy, a highly conserved eukaryotic cellular recycling process, plays an important role in promoting tumorigenesis and antitumor therapy. Many recent studies have linked autophagy to TNBC progression. It was reported that TNBC tumors exhibit a higher level of autophagy than other breast cancer subtypes.^[^ ^3^ ^]^ Expressions of the autophagy‐related proteins including Beclin1, LC3A, and LC3B are higher in TNBC cells than that in the other breast cancer subtypes.^[^ ^3^ ^]^ Also, it has been demonstrated that inhibition of autophagy via genetic intervention or pharmacological inhibitors resulted in suppression of cell stemness, proliferation, and metastasis with induction of apoptosis and inhibition of proto‐oncogenic pathways in TNBC cells.^[^ ^4^ ^]^ Autophagy has also been shown to play an essential role in promoting drug resistance of TNBC. Paclitaxel‐resistant TNBC cell lines had higher levels of autophagy than the parental cell lines when exposed to starvation.^[^ ^5^ ^]^ Moreover, there are evidences showing that autophagy inhibition restored the sensitivity of TNBC cells to chemotherapeutic agents.^[^ ^6^ ^]^ Therefore, autophagy has been appreciated as a promising therapeutic target for treatment of TNBC.

Long noncoding RNAs (lncRNAs) are loosely defined as RNAs that exceed 200 bases in length and have no apparent coding capacity, which have been shown to have important roles in promoting tumor formation and progression.^[^ ^7^, ^8^ ^]^ Currently, only few lncRNAs have recently been reported to be implicated in the modulation of autophagy in TNBC cells.^[^ ^4^ ^]^ For example, NAMPT‐AS is an oncogenic lncRNA in TNBC that epigenetically activates NAMPT to promote tumor progression and metastasis.^[^ ^9^ ^]^ Moreover, lncRNA OTUD6B‐AS1 promotes paclitaxel resistance in TNBC by regulation of miR‐26a‐5p/MTDH pathway‐mediated autophagy.^[^ ^10^ ^]^ As only a few autophagy‐associated lncRNAs were discovered in TNBC, the landscape of lncRNAs dysregulated and their molecular mechanisms in autophagic regulatory networks in TNBC remain largely unknown. Therefore, exploiting the newly emerging knowledge of the lncRNA‐autophagy‐cancer axis may provide novel targets and strategy for TNBC therapy.

Combination of therapeutic interventions provides patients with the opportunity to derive maximum benefit from therapy while minimizing or eliminating recurrence, resistance, and toxic effects.^[^ ^11^ ^]^ A growing number of studies have suggested that siRNA is an important RNA interference tool applied in cancer treatment, providing clinically translation potential for targeting oncogenic lncRNAs.^[^ ^12^ ^]^ Moreover, combining siRNA and other therapeutic agents can overcome drug resistance by simultaneously silencing genes and enhancing chemotherapeutic activity. Codelivery of these diverse anticancer agents, however, requires specially designed nanocarriers, such as liposomes, polymer‐based nanoparticles, and inorganic nanoparticles.^[^ ^13^ ^]^ Remarkably, our previous studies have successfully developed active targeting nanomedicines based on metal–organic frameworks (MOFs) for combination of siRNA with chemotherapeutics, providing an efficacious strategy for enhanced tumor therapy.^[^ ^14^, ^15^ ^]^

In the current study, using RNA sequencing approach we identified a new autophagy‐regulatory lncRNA, DNA damage inducible transcript 4 antisense RNA1 (DDIT4‐AS1), which is highly expressed in TNBC. We show that DDIT4‐AS1 activated autophagy through binding to both DDIT4 mRNA and the RNA binding protein AUF1, enhancing DDIT4 mRNA stability, resulting in the inhibition of mTOR signaling. We further demonstrated that the siRNA‐mediated silencing of DDIT4‐AS1 could sensitize TNBC cells to chemotherapeutic drugs, and codelivery of DDIT4‐AS1‐targeted siRNA and paclitaxel using our metal–organic frameworks (MOFs) nanoparticle‐based system significantly enhanced the efficacy of the chemotherapeutic agent in vitro, in vivo and patient‐derived organoids. This study implies that targeting of lncRNA DDIT4‐AS1 may be explored as a promising strategy to sensitive the TNBC response to chemotherapy.

## 2 Results

### 2.1 Identification of lncRNA DDIT4‐AS1 as an Autophagy Activator in TNBC Cells

**Figure 1:**
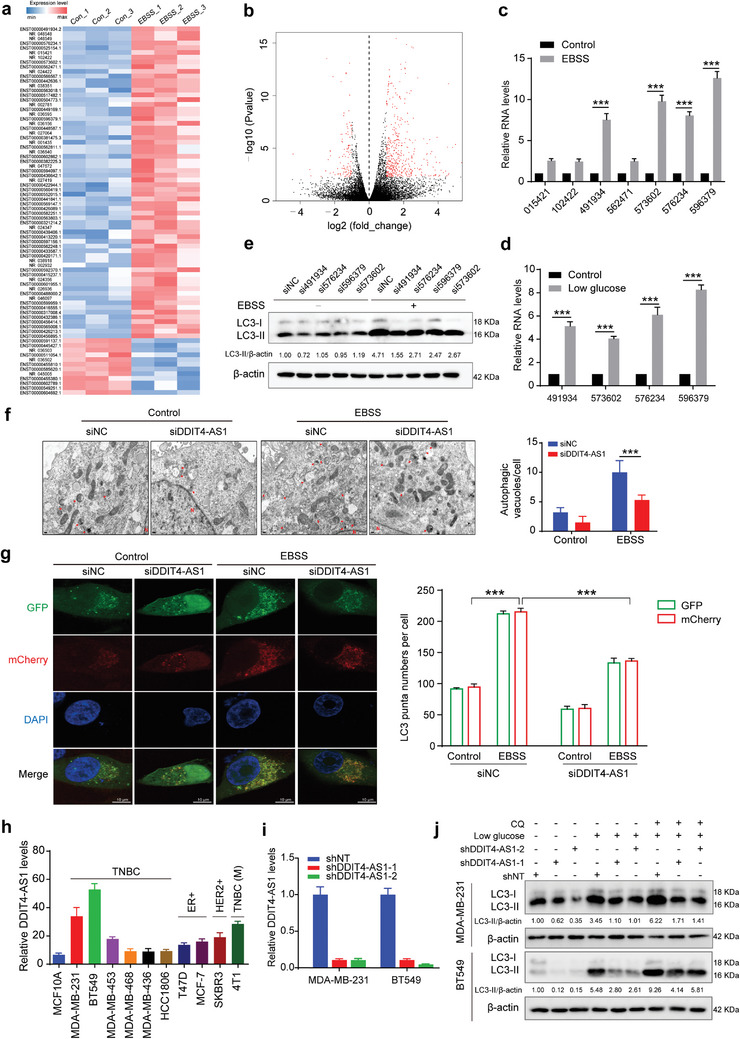
Identification of lncRNA DDIT4‐AS1 as an important regulator of autophagy in TNBC cells. a) Heat maps showing the differentially expressed lncRNAs between control and EBSS‐treated MDA‐MB‐231 cells by limiting the difference ratio (|log2 (Fold change)|>1) and significance level (*q*_value<0.05). Three samples were used in each group. Colors correspond to the expression level indicated by the log2‐transformed scale bar below the matrix. Red and blue reflect Max and Min levels, respectively. b) Volcano plots showing the expression profiles of lncRNAs (up 460, down 117). c,d) MDA‐MB‐231 cells were treated with EBSS for 6 h and low glucose (LG) medium for 24 h, respectively, and the expression levels of lncRNAs were detected via qRT‐PCR analysis. *β*‐actin was the internal control. ***p < 0.001. e) Western blot showing the effects of lncRNAs silencing on LC3‐II/*β*‐actin levels in MDA‐MB‐231 cells treated with EBSS. f) Representative electron microscopy images and quantification of autophagic vacuoles in DDIT4‐AS1 silencing MDA‐MB‐231 cells treated with EBSS. Scale bar, 2 µm. ***p < 0.001. Arrows depict autophagosomes, and the nucleus is denoted by N. g) Confocal microscopy showing the effects of EBSS incubation on mCherry‐GFP‐LC3 dots distribution in MDA‐MB‐231 DDIT4‐AS1 knockdown cells 48 h after mCherry‐GFP‐LC3 plasmid transfection (scar bar: 10 µm), ***p < 0.001. h) qRT‐PCR was performed to measure the differential expression of DDIT4‐AS1 in multiple breast cancer cells and the mammary epithelial cell MCF10A. TNBC cells: MDA‐MB‐231, BT549, MDA‐MB‐453, MDA‐MB‐468, MDA‐MB‐436, HCC1806; ER positive cells: T47D, MCF‐7; HER2 positive cells: SKBR3; murine TNBC: 4T1. *β*‐actin was the internal control. i) MDA‐MB‐231 and BT549 cell lines with stable DDIT4‐AS1 silencing were constructed using two sequences, respectively. j) Western blot showing the effects of DDIT4‐AS1 knockdown on LC3‐II/*β*‐actin levels in MDA‐MB‐231 and BT549 cells treated with chloroquine (CQ).

To identify the lncRNAs involved in the regulation of autophagy in TNBC cells, we first stressed the TNBC cell line MDA‐MB‐231 in the Earle's balanced salt solution (EBSS) to activate autophagy (activation of autophagy was confirmed by the elevated LC3‐II/LC3‐I ratio) (Figure S1a, Supporting Information), then conducted lncRNA sequencing. As shown in the Volcano plot and the Heat map (**Figure** **1**a,b), 577 differentially expressed lncRNAs were identified in the EBSS group compared to the control group, among them 460 were up‐regulated and 117 were down‐regulated. The seven significantly up‐regulated lncRNAs (Table S1, Supporting Information) were subsequently subjected to verification. We found that four lncRNAs, 491934, 573602, 576234, and 596379, were significantly up‐regulated in MDA‐MB‐231 cells following EBSS starvation (Figure 1c) or cultured in the low glucose medium (Figure 1d; and Figure S1b, Supporting Information). To demonstrate the roles of lncRNAs in autophagy, we examined the effects of silencing of the expressions of the lncRNA 491934, 573602, 576234, and 596379 on starvation‐induced autophagy. Figure 1e; and Figure S1c (Supporting Information) show that silencing of the lncRNA 491 934 significantly reduced the amount of LC3‐II in MDA‐MB‐231 cells treated with EBSS, compared to the other three lncRNAs, suggesting that lncRNA 491 934 is an activator of autophagy. The Ensemble database shows that lncRNA 491934 (ENST00000491934.2) is the transcript of gene *RP11‐442H21.2*, which is located at chromosomal band 10q22.1 and consists of two exons with a full length of 847 nt.^[^ ^17^ ^]^ In addition, 491934 is named as DDIT4‐AS1 because it is a single antisense lncRNA transcribed from the reverse strand of the DDIT4 locus. Electron microscopy examination observed that DDIT4‐AS1 knockdown reduced the numbers of autophagic vesicles (Figure 1f). Knockdown of DDIT4‐AS1 also significantly blunted the induction of autophagy, as evidenced by decreased autophagosomes labeled with yellow (mCherry^+^GFP^+^) and autolysosomes labeled with red (mCherry^+^GFP^−^) examined by confocal microscopy (Figure 1g). Examination of the expression of DDIT4‐AS1 in multiple breast cancer cell lines and the mammary epithelial cell line MCF10A found that DDIT4‐AS1 was highly expressed in TNBC cell lines MDA‐MB‐231 and BT549 cells (Figure 1h). Two shRNAs were used to successfully knockdown DDIT4‐AS1 in these two breast cancer cell lines (Figure 1i). We then investigated the effect of DDIT4‐AS1 on autophagic flux, and found that DDIT4‐AS1 depletion significantly decreased the level of LC3‐II in the presence or absence of chloroquine (CQ) in MDA‐MB‐231 and BT549 cells cultured in low glucose medium (Figure 1j). These results support DDIT4‐AS1 as a regulator of autophagy in TNBC cells.

### 2.2 High Expression of lncRNA DDIT4‐AS1 in TNBC Cells is Caused by its H3K27 Acetylation

**Figure 2:**
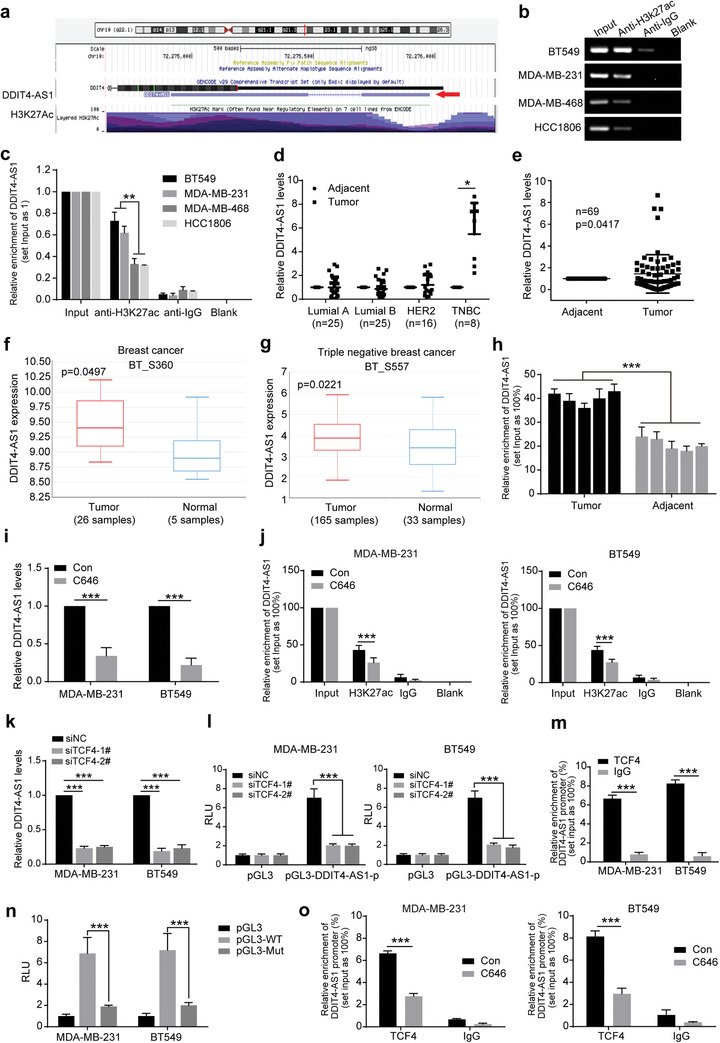
The lncRNA DDIT4‐AS1 is activated by H3K27ac. a) The gene annotation information in UCSC database indicated H3K27 acetylation of DDIT4‐AS1 promoter. b,c) CHIP assay using anti‐H3K27ac antibody showed that H3K27ac was enriched at the promoter region of DDIT4‐AS1. Moreover, the enriched level was significantly increased in BT549 and MDA‐MB‐231 cells in contrast to MDA‐MB‐468 and HCC1806 cells. d) The levels of DDIT4‐AS1 in different types of breast tumor and adjacent tissues were measured by qPCR. *β*‐actin was the internal control. *p < 0.05. e) The expression level of DDIT4‐AS1 in breast tumor and adjacent tissues was measured by using qPCR. *β*‐actin was the internal control. f) The relative expression of DDIT4‐AS1 in breast tumor and adjacent tissues was obtained from the LnCAR database (https://lncar.renlab.org/). g) The relative expression of DDIT4‐AS1 in TNBC and adjacent tissues was obtained from the LnCAR database. h) The H3K27ac enrichment was measured by ChIP experiment in 5 pair of TNBC tumor and adjacent tissues. i) DDIT4‐AS1 expression was measured by qPCR in breast cancer cells treated with C646 or Solvent control (Con) for 48 h. ***p < 0.001. j) C646 significantly suppressed the binding level of H3K27ac to DDIT4‐AS1 promoter. ***p < 0.001. k) MDA‐MB‐231 and BT549 cell lines were transfected with TCF4 siRNA, then the level of DDIT4‐AS1 was measured by qPCR. *β*‐actin was the internal control. ***p < 0.001. l) Detection of luciferase activity in DDIT4‐AS1 promoter in MDA‐MB‐231 and BT549 cells after knockdown of TCF4. ***p < 0.001. m) CHIP‐qPCR assay using anti‐TCF4 antibody. ***p < 0.001. n) Breast cancer cells were transfected with the plasmids for 2 d. The luciferase activity levels were normalized to pGL3 luciferase activity. ***p < 0.001. o) CHIP‐qPCR assay using anti‐TCF4 antibody. ***p < 0.001.

Next, we wanted to understand how the expression of DDIT4‐AS1 is highly expressed in TNBC cells. As recent studies have shown that aberrant expression of lncRNAs may be associated with acetylation mediated‐transcriptional activation,^[^ ^18^, ^19^, ^20^, ^21^ ^]^ we investigated the probable epigenetic modification of DDIT4‐AS1 using genome bioinformatics analysis (http://genome.ucsc.edu/), and found that the promoter region of DDIT4‐AS1 has high enrichment of H3K27ac (**Figure** **2**a; and Figure S2a, Supporting Information). ChIP assay showed that the H3K27ac was enriched at the promoter region of DDIT4‐AS1 gene in the TNBC cell lines (Figure 2b). Moreover, there is higher H3K27ac enrichment in the promoter of DDIT4‐AS1 in BT549 and MDA‐MB‐231 cells with high DDIT4‐AS1 expression than that in HCC1806 cells and MDA‐MB‐468 cells with low DDIT4‐AS1 expression (Figure 2c). We further observed that TNBC specimens exhibited higher expression of DDIT4‐AS1 than other subtypes of breast cancer (Figure 2d). DDIT4‐AS1 expression was aberrantly up‐regulated in tumor tissues compared to normal tissues (Figure 2d,e), which was consistent with the analyses of the online dataset (Figure 2f,g). As expected, there are increased levels of DDIT4‐AS1‐H3K27ac in the TNBC tissues compared to normal tissues (Figure 2h). Further, the histone acetyltransferase (HAT) inhibitor C646 significantly repressed the DDIT4‐AS1 expression and its H3K27ac level in BT549 and MDA‐MB‐231 cells (Figure 2i,j). These results indicate that the up‐regulation of DDIT4‐AS1 is induced by its histone acetylation at the promoter region. In addition, we evaluated the potential correlation between DDIT4‐AS1 expression and clinicopathological characteristics of breast cancer patients, and found that DDIT4‐AS1 was positively associated with the TNM stage (*p* = 0.0116) and Ki67 staining (*p* = 0.0147, Table S2, Supporting Information). The survival probability analysis revealed that DDIT4‐AS1 expression was associated with overall survival (OS) rates of breast cancer patients (Figure S2b, Supporting Information).

Next, we queried which transcription factor bind to DDIT4‐AS1 after the promoter region is modified by H3K27ac. Online prediction results showed that TCF4, ASCL1, and KLF17 may be transcription factors of DDIT4‐AS1 using the JASPAR TFBS in the UCSC Genome Browser (Figure S2c, Supporting Information). Then, we used the JASPAR database to predict the binding site sequences of the candidate transcription factors in the promoter regions of DDIT4‐AS1 (Figure S2d, Supporting Information). Considering that TCF4 is an important oncogene in breast cancer and has a highest score, we therefore selected TCF4 for further verification. Silencing TCF4 greatly reduced DDIT4‐AS1 level and the luciferase activity of DDIT4‐AS1 promoter in both MDA‐MB‐231 and BT549 cells (Figure 2k,l, Figure S2e, Supporting Information). ChIP‐qPCR experiments showed that TCF4 was enriched at the promoter region of DDIT4‐AS1 (Figure 2m; and Figure S2f, Supporting Information). Moreover, we designed the wild‐type (pGL3‐WT) and mutant (pGL3‐Mut) luciferase reporter vectors based on the TCF4 binding site at DDIT4‐AS1 promoter (CTGCACCTGCCTG). The luciferase activity of pGL3‐WT was significantly increased in MDA‐MB‐231 and BT549 cells, whereas the luciferase activity of pGL3‐Mut‐transfected cells was significantly decreased (Figure 2n). These results indicate that TCF4 is a transcription factor of DDIT4‐AS1. Further, we observed that C646 treatment reduced the enrichment of TCF4 on DDIT4‐AS1 promoter (Figure 2o), suggesting that H3K27ac modification is necessary for TCF4‐mediated transcriptional activation of DDIT4‐AS1.

### 2.3 The lncRNA DDIT4‐AS1 Stabilizes DDIT4 mRNA by Recruiting AUF1

**Figure 3:**
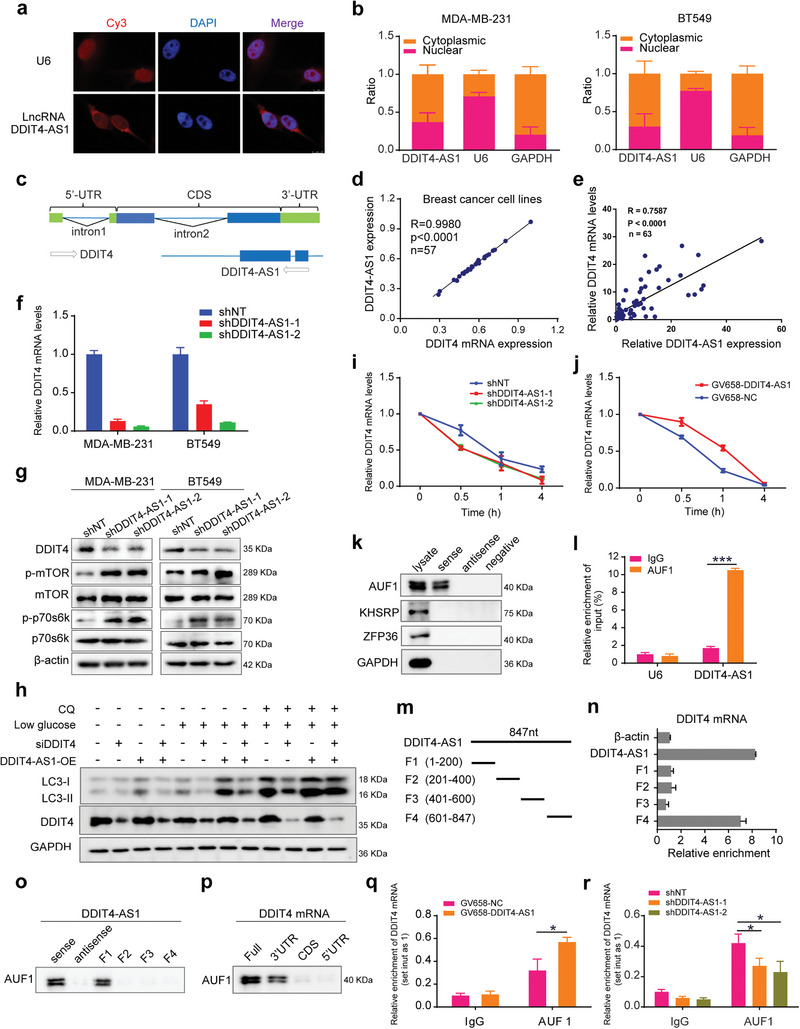
LncRNA DDIT4‐AS1 regulates stability of DDIT4 mRNA via recruiting AUF1. a) The location of lncRNA DDIT4‐AS1 in MDA‐MB‐231 cells was determined by FISH assay. Cy3 labeled probes are red; DAPI‐stained nuclei are blue; and U6 served as the positive control (scale bar: 10 µm). b) The expression level of DDIT4‐AS1 in the subcellular fractions of MDA‐MB‐231 and BT549 cells was detected by qRT‐PCR. U6 and GAPDH were used as nuclear and cytoplasmic markers, respectively. c) DDIT4 is a protein encoding gene adjacent to DDIT4‐AS1 locus. Location information was obtained from the UCSC Genome Browser. d) The correlation of DDIT4‐AS1 and DDIT4 mRNA was analyzed using the relative expression level from CLEE dataset. e) QRT‐PCR was used to detect he expression of DDIT4‐AS1 and DDIT4 mRNA in 63 paired breast tumor/adjacent tissues. *β*‐actin used as the internal control. f) QRT‐PCR was used to detect the effect of DDIT4‐AS1 knockdown on the expression of DDIT4 in breast cancer cells. *β*‐actin was the internal control. g) Western blot was used to detect the effect of DDIT4‐AS1 knockdown on the expression of indicated proteins in breast cancer cells. h) Western blot showing LC3‐II/LC3‐I and DDIT4 levels in MDA‐MB‐231 cells under treatment. i) 2.5 µg mL^−1^ ActD were used to block mRNA transcription, and qRT‐PCR was used to detect the effect of DDIT4‐AS1 knockdown on the degradation rate of DDIT4 mRNA in MDA‐MB‐231. 18s was the internal control. j) 2.5 µg mL^−1^ ActD were used to block mRNA transcription. qRT‐PCR was used to detect the effect of DDIT4‐AS1 overexpression on the degradation rate of DDIT4 mRNA in MDA‐MB‐231. 18s was the internal control. k) RNA pull down followed by western blotting was done with DDIT4‐AS1 probe to verify the direct interaction between RBPs and DDIT4‐AS1 in MDA‐MB‐231. l) In MDA‐MB‐231 cells, RIP was performed using anti‐AUF1 and control IgG antibodies, followed by qRT‐PCR to examine the enrichment of DDIT4‐AS1 and U6. U6 served as negative control, ***p < 0.001. m) The schematic of full‐length, truncated DDIT4‐AS1 (F1‐F4). n) The interaction between truncated DDIT4‐AS1 and DDIT4 mRNA, shown by RNA pull‐down and qRT‐PCR in MDA‐MB‐231. o,p) RNA pull‐down and western blotting showing the interaction of full‐length and truncated DDIT4‐AS1 o) or DDIT4 mRNA p) with AUF1 in MDA‐MB‐231 cell lysates. q,r) RIP was performed to detect endogenous AUF1 binding to DDIT4 mRNA in MDA‐MB‐231 cells with DDIT4‐AS1 overexpression q) or knockdown r). *p <0.05.

We then sought to identify the downstream effectors involved in the regulation of autophagy by DDIT4‐AS1. First, we demonstrated that DDIT4‐AS1 located primarily in the cytoplasm using confocal microscopy for fluorescent in situ hybridization (FISH) and nuclear/cytoplasm fractionation (**Figure** **3**a,b), suggesting that DDIT4‐AS1 may primarily exert its biological function in the cytoplasm. Certain mammalian lncRNAs are embedded in the intronic‐antisense regions of protein‐coding genes and regulate the parental genes to exert their function.^[^ ^7^, ^9^, ^22^ ^]^ Thus, we asked whether there is a functional relationship between DDIT4‐AS1 and the corresponding protein‐coding gene DDIT4 (Figure 3c). By analyzing the RNA sequencing data, we found that the levels of DDIT4‐AS1 and DDIT4 mRNA were both up‐regulated after EBSS treatment (Table S3, Supporting Information). The positive correlation between the levels of DDIT4‐AS1 and DDIT4 mRNA were verified in 57 breast cancer cell lines from CCLE dataset as well as 63 pairs of breast tumor tissues (Figure 3d,e). We further observed that DDIT4 expression was aberrantly higher in basal like breast tumors as compared to other subtypes of breast cancer (Figure S3a, Supporting Information). In MDA‐MB‐231 and BT549 cells with DDIT4‐AS1 knockdown, the expressions of DDIT4 mRNA and protein were significantly down‐regulated (Figure 3f,g). It is known that DDIT4 can activate autophagy by inhibiting mTORC1.^[^ ^23^, ^24^ ^]^ Here, we found that silencing DDIT4‐AS1 up‐regulated the expressions of p‐mTOR and p‐p70s6k (Figure 3g). DDIT4‐AS1‐induced autophagy was reversed by DDIT4 knockdown in MDA‐MB‐231 cells with starvation (Figure 3h; and Figure S3b, Supporting Information). These data suggest that lncRNA DDIT4‐AS1 activates autophagy by regulating DDIT4‐mTOR signaling pathway.

We next wanted to explore how DDIT4‐AS1 regulates DDIT4 mRNA. Given the cytoplasmic localization of DDIT4‐AS1, we hypothesized that DDIT4‐AS1 may affect DDIT4 expression at posttranscriptional level. To test this hypothesis, we first measured the levels of DDIT4 pre‐mRNA and mature mRNA upon in the cells with DDIT4‐AS1 disruption. Figure S3c,d (Supporting Information) shows that the levels of DDIT4 mature mRNA (3’‐UTR, CDS (coding sequence) and 5’‐UTR) were significantly decreased or increased when DDIT4‐AS1 was downregulated or upregulated, but the level of DDIT4 pre‐mRNA containing two intronic regions (intron‐1 and intron‐2) remained unchanged, suggesting that DDIT4‐AS1 does not affect the transcription of DDIT4 but may post‐transcriptionally regulate DDIT4 mRNA expression. We then carried out RNA stability assay using actinomycin D (ActD) to block mRNA transcription, and observed that knockdown of DDIT4‐AS1 accelerated the degradation of DDIT4 mRNA, while ectopic expression of DDIT4‐AS1 evidently increased the half‐life of DDIT4 mRNA (Figure 3i,j). These results indicate that DDIT4‐AS1 elevates DDIT4 through maintaining its mRNA stability.

RNA binding proteins (RBPs) bind cis‐regulatory elements in the 3' untranslated regions (UTRs) of mRNA and regulate mRNA turnover and translation.^[^ ^25^ ^]^ lncRNAs can recruit RBPs to regulate the stability of downstream mRNA molecules.^[^ ^26^, ^27^, ^28^ ^]^ To identify the potential RBPs associated with both of DDIT4‐AS1 and DDIT4 mRNA, we performed online analysis of binding capacity between DDIT4‐AS1 or DDIT4 mRNA with several classic RBPs, and picked three (AUF1, ZFP36, KHSRP) with higher scores for validation (Table S4, Supporting Information). QRT‐PCR assay showed that silencing AUF1 significantly decreased DDIT4 mRNA level, but silencing KHSRP or ZFP36 had no effect on DDIT4 mRNA level (Figure S3e,f, Supporting Information). Further, RNA stability assay showed that silencing AUF1 instead of KHSRP or ZFP36 abolished the accelerated degradation of DDIT4 mRNA caused by DDIT4‐AS1 knockdown (Figure S3g, Supporting Information), suggesting that AUF1 was required for DDIT4‐AS1‐induced higher stability of DDIT4 mRNA. RNA pull‐down assay further verified that AUF1, but not KHSRP or ZFP36, was enriched by biotinylated DDIT4‐AS1 (Figure 3k). RIP assay verified that DDIT4‐AS1 was precipitated by AUF1 antibody (Figure 3l). These results collectively suggested that DDIT4‐AS1 binds with the RBP AUF1, which may be account for the stability regulation of DDIT4 mRNA.

Because there are some overlap gene sequences of DDIT4‐AS1 and DDIT4, we performed sequence alignment analysis, which indicates that DDIT4‐AS1 might directly interact with the DDIT4 mRNA. To determine which fragment of DDIT4‐AS1 is responsible for the interaction with DDIT4 mRNA or AUF1 protein, respectively, we fragmented DDIT4‐AS1, then performed RNA pull‐down assay (Figure 3m). Figure 3n shows that the DDIT4‐AS1 fragment 4 (601–847 nt) exhibited the highest binding affinity to DDIT4 mRNA. The fragment 1 consisting of bases 1–200 of DDIT4‐AS1 was crucial for the interaction with AUF1, whereas other fragments did not appear to interact with AUF1 (Figure 3o). It is known that lncRNAs recruit RBPs to 3’UTR of mRNA transcripts in order to modulate the RNA stability. Therefore, we assayed the physical interaction between DDIT4 mRNA and AUF1, and observed that AUF1 specifically associates with biotin‐labeled full length or 3’UTR but not 5’UTR or CDS of DDIT4 mRNA (Figure 3p). Hence, our results proved that DDIT4‐AS1 recruits AUF1 to the DDIT4 3’UTR to stabilize DDIT4 mRNA.

It has to be noted that AUF1 silencing did not affect the expression of DDIT4‐AS1; in turn, the protein levels of AUF1 were not affected by DDIT4‐AS1 knockdown (Figure S3h,i, Supporting Information). To determine whether DDIT4‐AS1 affects the binding of AUF1 to DDIT4 mRNA, we performed RIP assay and observed that overexpression of DDIT4‐AS1 enhanced the binding of AUF1 to DDIT4 mRNA while knockdown of DDIT4‐AS1 exerted an opposite effect (Figure 3q,r). Furthermore, we demonstrated that silencing of AUF1 abrogated the up‐regulation of DDIT4 mRNA induced by DDIT4‐AS1 overexpression (Figure S3j, Supporting Information). Taken together, these data demonstrate that DDIT4‐AS1 enhances the interaction between AUF1 and DDIT4 mRNA, and that the regulation of DDIT4‐AS1 on DDIT4 mRNA is mainly dependent on AUF1.

### 2.4 DDIT4‐AS1 Promotes the Proliferation and Migration of TNBC Cells via Activating Autophagy

**Figure 4:**
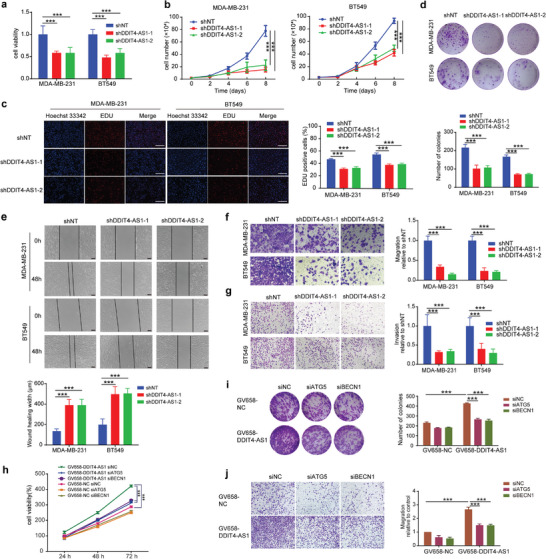
Knockdown of DDIT4‐AS1 inhibits proliferation and migration of breast cancer cells. a) After 48 h of cell seeding, CCK8 assay revealed that knockdown of DDIT4‐AS1 reduced cell viability of MDA‐MB‐231 and BT549. ***p < 0.001. b) The effects of DDIT4‐AS1 knockdown on the proliferation of MDA‐MB‐231 and BT549 cells were examined by cell counting assay. ***p < 0.001. c) EDU assays were used to detect the proliferation rate of MDA‐MB‐231 and BT549 cells after DDIT4‐AS1 knockdown. Scale bar, 200 µm. ***p < 0.001. d) The effects of lncRNA DDIT4‐AS1 knockdown on the proliferation of MDA‐MB‐231 and BT549 cells were examined by colony formation assays. ***p < 0.001. e) Scratch assay was done to show the effect of DDIT4‐AS1 knockdown on cell migration ability. Scale bar, 100 µm. ***p < 0.001. f) Representative images and quantitative analysis of transwell migration assay showing that downregulation of DDIT4‐AS1 decreased cell migratory ability. Magnification, × 200. ***p < 0.001. g) Representative images and quantitative analysis of transwell invasion assay showing that downregulation of DDIT4‐AS1 decreased cell invasive ability. Magnification, × 100. ***p < 0.001. h) CCK8 assay was used to reveal cell viability of MDA‐MB‐231 cell with various treatments. ***p < 0.001. i) Colony formation assay was used to examine the proliferation of MDA‐MB‐231 cells subjected to various treatments. ***p < 0.001. j) Trans‐well assay was used to reveal migration of MDA‐MB‐231 cell subjected to various treatments. Magnification, × 100. ***p < 0.001.

We next determined the essential role of DDIT4‐AS1 in the progression of TNBC, and found that knockdown of DDIT4‐AS1 significantly repressed the cell viability of MDA‐MB‐231 and BT549 cells (**Figure** **4**a,b). In addition, there are decreased EDU positive cells (representing mitotic S phrase cells) in cells with DDIT4‐AS1 knockdown (Figure 4c). Furthermore, colony formation ability was decreased in TNBC cells with DDIT4‐AS1 silencing (Figure 4d). These results demonstrated that DDIT4‐AS1 promotes cell proliferation in TNBC cells. We further investigated the effect of DDIT4‐AS1 on migration and invasion of TNBC cells. As shown in Figure 4e–g, silencing of DDIT4‐AS1 significantly inhibited cell motility, migration and invasion in MDA‐MB‐231 and BT549 cells. Collectively, these data suggest that DDIT4‐AS1 promotes cell proliferation and migration, acting as an oncogene in TNBC.

To investigate whether autophagy is involved in the regulation of DDIT4‐AS1 in promoting tumor progression, MDA‐MB‐231 cells were transfected with GV658‐DDIT4‐AS1 plasmid and BECN1 siRNA or ATG5 siRNA (Figure S4a,b, Supporting Information), and cell viability as well as migration was measured. As shown in Figure 4h,i; and Figure S4c (Supporting Information), the increased cell proliferation ability induced by overexpression of DDIT4‐AS1 was significantly abolished by silencing of BECN1 or ATG5. Consistently, the increase in cell migration rate induced by DDIT4‐AS1 overexpression was also blocked by BECN1 or ATG5 knockdown, as examined by scratch and trans‐well migration assays (Figure S4d (Supporting Information); and Figure 4j). Similarly, the increased cell viability and migration induced by up‐regulation of DDIT4‐AS1 were attenuated in the cells treated with the autophagy inhibitors CQ or 3‐MA (3‐methyladenine) (Figure S4e–h, Supporting Information). In contrast, the restoration of cell growth and migration were observed when autophagy inducer (rapamycin, rapa) was used after knockdown of DDIT4‐AS1 (Figure S4i,j, Supporting Information). Taken together, these data indicate that DDIT4‐AS1 promotes TNBC cell proliferation and migration through activating autophagy.

### 2.5 Silencing of DDIT4‐AS1 Enhances the Chemosensitivity of TNBC Cells to Paclitaxel

**Figure 5:**
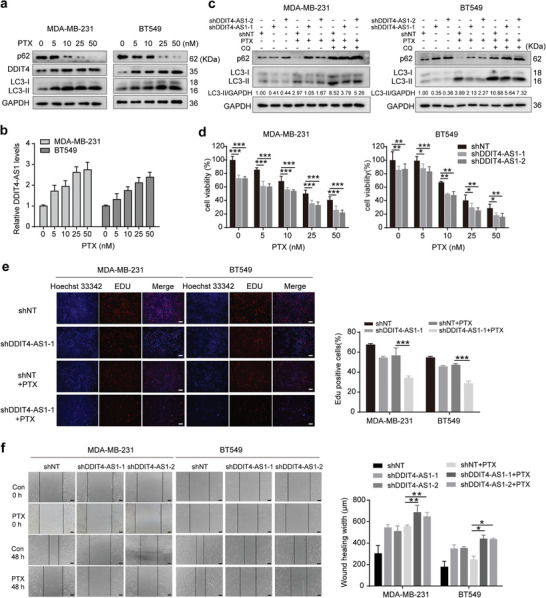
DDIT4‐AS1 attenuates chemosensitivity of breast cancer cells to paclitaxel. a) MDA‐MB‐231 and BT549 cells were treated with paclitaxel (PTX) as indicated, and then the level of LC3, p62, and DDIT4 proteins was examined by western blot. b) MDA‐MB‐231 and BT549 cells were treated with PTX as indicated, and then the level of DDIT4‐AS1 was examined by qRT‐PCR. *β*‐actin was the internal control. c) Western blot showing the effects of DDIT4‐AS1 knockdown on the expressions of LC3‐II/LC3‐I and p62 in MDA‐MB‐231 and BT549 cells treated with PTX (25 nM) and chloroquine (CQ). d) Cell viability was analyzed by CCK‐8 assay after PTX treatment for 48 h in DDIT4‐AS1 knockdown cells. *p < 0.05, **p < 0.01, ***p < 0.001. e) EDU assays were used to detect the proliferation rate of DDIT4‐AS1 knockdown cells after 25 nM PTX treatment for 48 h. Scale bar, 100 µm. ***p < 0.001. f) Scratch assay was done to show the migration ability of DDIT4‐AS1 knockdown MDA‐MB‐231 and BT549 cells treated with 25 nM PTX for 24 h. Scale bar, 100 µm. *p < 0.05. **p < 0.01.

**Figure 6:**
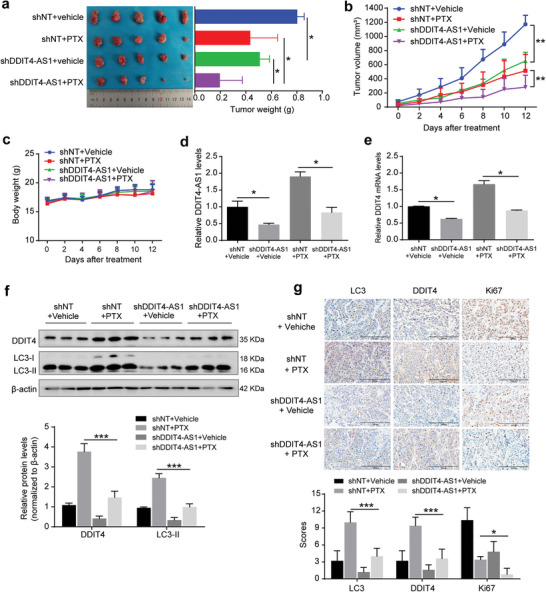
Downregulation of DDIT4‐AS1 improves sensitivity of breast cancer cells to paclitaxel in vivo. a,b) MDA‐MB‐231 cells with stable expression of a DDIT4‐AS1‐targeted shRNA or a nontargeting shRNA were subcutaneously injected into nude mice. The mice were randomly divided into four groups: shNT+vehicle, shNT+ paclitaxel, shDDIT4‐AS1+vehicle, shDDIT4‐AS1+PTX, and 10 mg kg^−1^ PTX was given intraperitoneally once every 3 days, and tumor volumes were measured on the days as indicated. After 2 weeks, the mice were sacrificed and tumor weights were examined. The values are presented as mean ± s.d. (*n* = 5), *p < 0.05, **p < 0.01; two‐way ANOVA. c) The effect of treatment on mice body weight. d,e) qPCR analysis of DDIT4‐AS1 and DDIT4 mRNA expression in the MDA‐MB‐231 xenografts following the indicated treatment. *β*‐actin was the internal control. *p < 0.05. f) WB analysis of the indicated protein expression in the MDA‐MB‐231 xenografts following the indicated treatment. ***p < 0.001. g) Representative Ki67, DDIT4, and LC3 staining and quantitative analysis of orthotopic xenograft sections from DDIT4‐AS1 downregulation and control groups treated with or without PTX. Scale bar, 200 µm. *p < 0.05, ***p < 0.001.

It is known that activation of autophagy can modulate tumor cell sensitivity to chemotherapy. For instance, the microtubule‐disrupting agent paclitaxel can elicit an autophagic response that actually plays a protective role, impeding its antitumoral efficiency.^[^ ^29^, ^30^, ^31^ ^]^ Consistently, we found that the expressions of autophagy‐related proteins, LC3, ATG5, and BECN1 were increased in TNBC tissues as compared to other breast cancer tissues, or as compared to the normal tissues, confirming activated autophagy existing in human TNBC specimens (Figure S5a,b, Supporting Information). Bioinformatics analysis further showed that the high expressions of DDIT4 and autophagy‐related genes were associated with poor relapse‐free survival in breast cancer patients undergoing chemotherapy (Figure S5b–e, Supporting Information). We show that paclitaxel treatment up‐regulated the expressions of DDIT4 and DDIT4‐AS1 as well as activated autophagy in TNBC cells (**Figure** **5**a,b). Furthermore, we found that silencing DDIT4‐AS1 significantly inhibited the induction of autophagy and the activation of DDIT4‐mTOR signaling pathway in the tumor cells treated with paclitaxel (Figure 5c; and Figure S6a, Supporting Information). We next determined whether DDIT4‐AS1 affects the sensitivity of TNBC cells to paclitaxel through in vitro and in vivo experiments. As shown in Figure 5d–f, DDIT4‐AS1 knockdown significantly enhanced the sensitivity of TNBC cells to paclitaxel, as reflected by the reduced cell proliferation and migration ability. In addition, we found that treatment of other chemotherapeutic agents, such as cisplatin (DDP) and doxorubicin (DOX) also caused up‐regulation of the expressions of LC3‐II and DDIT4‐AS1, and silencing DDIT4‐AS1 decreased the expression of LC3‐II and enhanced the antitumor efficacy of DDP and DOX in MDA‐MB‐231 cells, suggesting that the protective autophagy induced by chemotherapy drugs is causally related to the up‐regulation of DDIT4‐AS1 (Figure S6b–e, Supporting Information). Moreover, DDIT4‐AS1 knockdown not only inhibited tumor growth, but also enhanced the cytotoxicity of paclitaxel in MDA‐MB‐231 cell xenografts (**Figure** **6**a,b). There was no substantial weight loss in the treated mice (Figure 6c). In addition, the cell proliferation was significantly suppressed in the DDIT4‐AS1 knockdown group, as measured by Ki67 staining of tumor sections (Figure 6g). Consistently, DDIT4‐AS1 knockdown inhibited the up‐regulation of DDIT4‐AS1, DDIT4, and LC3‐II in MDA‐MB‐231 xenografts triggered by paclitaxel treatment (Figure 6d–g). Collectively, these data demonstrate that knockdown of DDIT4‐AS1 can enhance the sensitivity of TNBC cells to paclitaxel by blocking autophagy.

### 2.6 Characterization of the Core–Shell Nanosystem to Codeliver Paclitaxel and DDIT4‐AS1 siRNA

**Figure 7:**
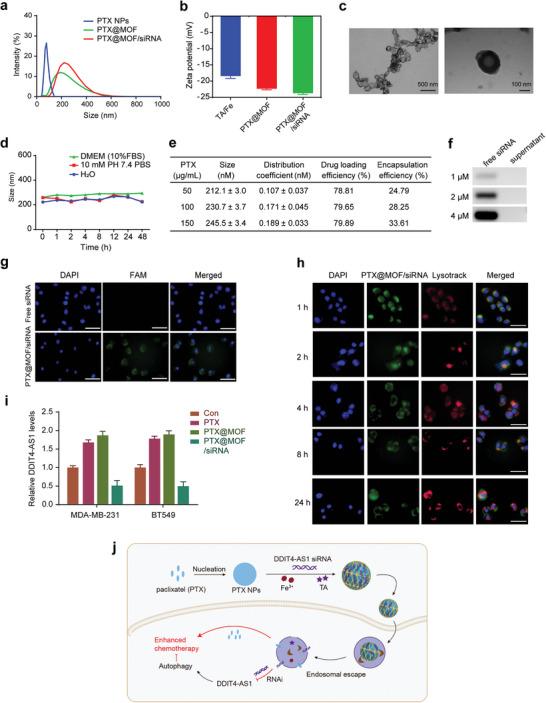
Characterization of the Core–Shell nanosystem codeliverying of paclitaxel and DDIT4‐AS1 siRNA. a) The particle size analysis of PTX NPs, PTX@MOF, and PTX@MOF/siDDIT4‐AS1. b) Zeta‐potential of TA/Fe^3+^, PTX@MOF, and PTX@MOF/siDDIT4‐AS1 (*n* = 3). c) TEM image images of PTX@MOF/siDDIT4‐AS1 nanoparticles. d) Storage stability investigation of PTX@MOF/siDDIT4‐AS1 (*n* = 3). e) Effects of PTX concentration on particle size, distribution coefficient, encapsulation rate, and drug loading of PTX@MOF/siRNA. f) PAGE images for characterization of DDIT4‐AS1 siRNA loading. g) Fluorescent images of MDA‐MB‐231 cells after incubating with free siDDIT4‐AS1 and PTX@MOF/siDDIT4‐AS1 for 4 h. Scale bar, 100 µm. h) Costaining of endo/lysosomes to evaluate the endo/lysosomes escape in MDA‐MB‐231 cells. Scale bar, 50 µm. i) qRT‐PCR was used to detect the effect of indicated treatments on the expression of DDIT4‐AS1 in breast cancer cells. *β*‐actin was the internal control. j) The preparation process of PTX@MOF/siDDIT4‐AS1 and its detailed mechanism for enhanced anticancer Therapy.

Based on the above experiments showing the benefits of DDIT4‐AS1 knockdown in sensitizing tumor cells to paclitaxel, we next designed and fabricated a nano‐based system that can codeliver paclitaxel and DDIT4‐AS1 siRNA to tumors. First, a pure paclitaxel nanocore was formed via solvent exchange, and the nanocore showed white and opalescent appearance, but was unstable and prone to aggregation within half an hour (Figure S7a, Supporting Information). To enhance the colloidal stability, the nanocore was rapidly coated with MOF shell upon its formation via TA/Fe^3+^ coordination, through which the siRNA of DDIT4‐AS1 was concomitantly encapsulated into the shell layer to form PTX@MOF/siDDIT4‐AS1 NPs according to our previous reports.^[^ ^14^, ^32^, ^33^ ^]^ After MOF coating, the dynamic size increased from 78 to 230 nm, and the *ζ* potential was −23.7 mV (**Figure** **7**a,b). The obtained PTX@MOF/siDDIT4‐AS1 showed a spherical core–shell structure with a diameter of 200 ± 24 nm (*n* = 7) and the shell thickness of 20 nm (Figure 7c) based on TEM microimages. The colloidal stability of PTX@MOF/siDDIT4‐AS1 was then tested, and no remarkable size change was observed within 48 h in both PBS buffer and cell culture medium (containing 10% FBS) (Figure 7d), indicating the high stability. The amount of paclitaxel loading was quantified by HPLC analysis, and the entrapment efficiency (EE%) and loading capacity (LC%) reached ≈79% and 28%, respectively (Figure 7e). To evaluate the encapsulation of siDDIT4‐AS1, gel electrophoresis was used to detect unloaded siRNA indirectly (supernatant) as compared to the corresponding free siRNA. The result showed a high loading efficiency of the MOF shell, achieving quantitative encapsulation with feeding siRNA concentration up to 4 µM (Figure 7f). Note that our nanosystem was rather simple, which only contained the biocompatible components of TA and Fe^3+^. However, its drug loading capacity is pretty high, thus presenting a promising nanoplatform for clinical translation.

The intracellular delivery of the shell–core nanosystem was then tested on MDA‐MB‐231 and BT549 cell lines. To measure cellular uptake, we incubated FAM‐siRNA‐encapsulated nanoparticles with the tumor cells, followed by fluorescence microscopy examination of the cells. FAM‐labeled free DDIT4‐AS1 siRNA did not show any green fluorescence inside cells due to the negatively charged nature of double strand RNA that is repelled by the cell membrane, while strong fluorescence was observed in the cells cultured with nanoparticles containing FAM‐siDDIT4‐AS1 (Figure 7g; and Figure S7b, Supporting Information). After transfection, most of the FAM‐siRNA (green fluorescence) resided in the endosome/lysosome (red fluorescence) of MDA‐MB‐231 cells at 1–2 h, as evidenced by colocalization of FAM‐siRNA and endosome/lysosome to produce orange signal (Figure 7h). However, at 4–8 h after transfection, a small portion of green fluorescence was separated from the red fluorescence. At 24 h after transfection, more separation between green and red fluorescence was observed (Figure 7h). The same fluorescence changes occurred in BT549 cells, but at slightly different times (Figure S7c, Supporting Information). These results are consistent with the previous studies showing that TA‐based MOF can promote lysosomal escape of nanoparticles through “proton‐sponge effect.”^[^ ^32^ ^]^ Superior uptake efficiency and easy escape from endosomal/lysosomal compartment regions can successfully promote gene silencing of nonviral gene vectors. As shown in Figure 7i, compared to the control group, all formulations containing paclitaxel alone exhibited conspicuous DDIT4‐AS1 upregulation. By contrast, the combined delivery of DDIT4‐AS1 siRNA and paclitaxel using nanoparticles resulted in significantly lower DDIT4‐AS1 expression (Figure 7i). Collectively, as summarized in Figure 7j, these results suggest that the codelivery nanoparticles have the capacity to facilitate the intracellular delivery and endosomal/lysosomal escape of payloads, releasing both paclitaxel and DDIT4 siRNA to exert their effects.

### 2.7 The Codelivery of the Nanoparticles Exert Strong Antitumor Effect on TNBC Cell, Xenografts, and Organoids Derived from Patient Samples

**Figure 8:**
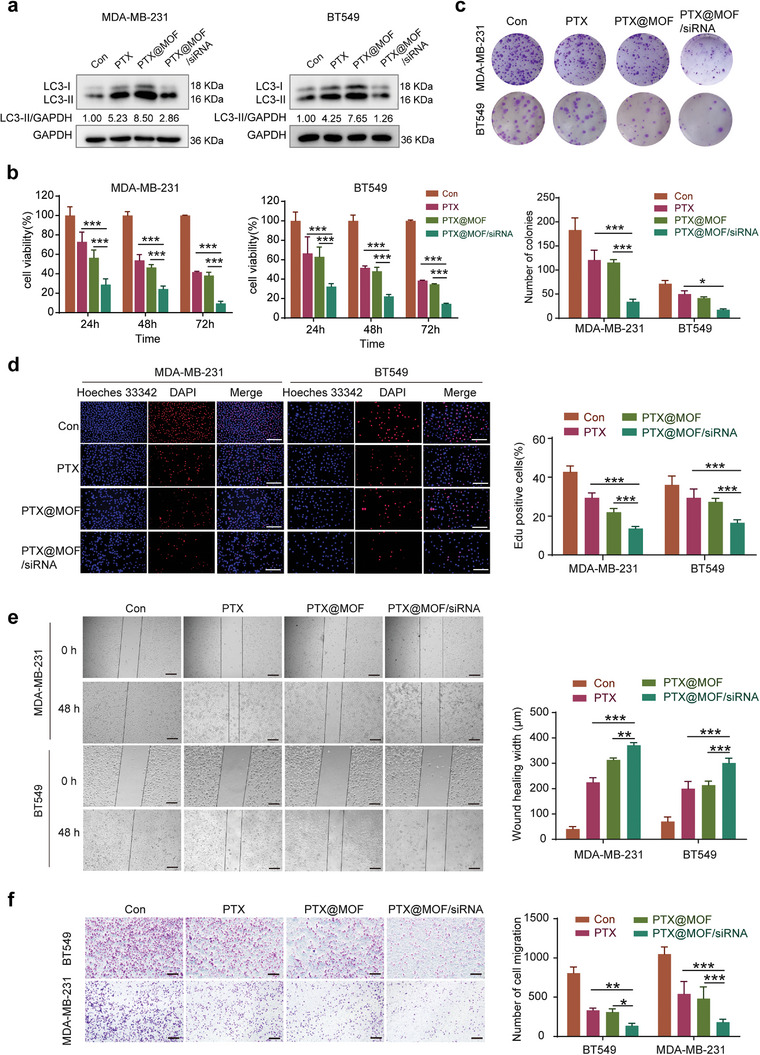
In vitro antitumor activity of the codelivery nanoparticles. a) WB was used to detect the effect of indicated treatments on the expression of LC3 in breast cancer cells. b) Cell viability was analyzed by CCK‐8 assay after indicated treatment in MDA‐MB‐231 cells. ***p < 0.001. c) Colony formation assays were carried out in MDA‐MB‐231 and BT549 cells. *p < 0.05, ***p < 0.001. d) EDU assays were used to detect the proliferation rate of MDA‐MB‐231 and BT549 cells after indicated treatments. Scale bar, 200 µm. ***p < 0.001. e) Scratch assay was done to show the migration ability of cells treated with different PTX preparations for 48 h. Scale bar, 200 µm. **p < 0.01, ***p < 0.001. f) Transwell migration assay showed cell migratory ability was affected by different PTX preparations. Scale bar, 200 µm. *p < 0.05, **p < 0.01, ***p < 0.001.

We first measured the effect of the nanoparticle on autophagy, and found that when the cells were treated with PTX@MOF/siDDIT4‐AS1, the expression of LC3‐II was much lower than that of the cells treated with the PTX or PTX@MOF alone (**Figure** **8**a), indicating that the PTX‐induced autophagy can be effectively inhibited via codelivery of DDIT4‐AS1 siRNA nanoparticles. As shown in Figure 8b–d, PTX@MOF/siDDIT4‐AS1 displayed stronger cell cytotoxicity than both free PTX and PTX@MOF. At the same time, the scratch and transwell assays demonstrated that PTX@MOF/siDDIT4‐AS1 markedly inhibited the migration ability of TNBC cells than both free PTX and PTX@MOF did (Figure 8e,f). These results show that PTX@MOF/siDDIT4‐AS1 is an excellent PTX delivery carrier and an efficient DDIT4‐AS1 silencer, which contribute to improved antitumor therapy.

**Figure 9:**
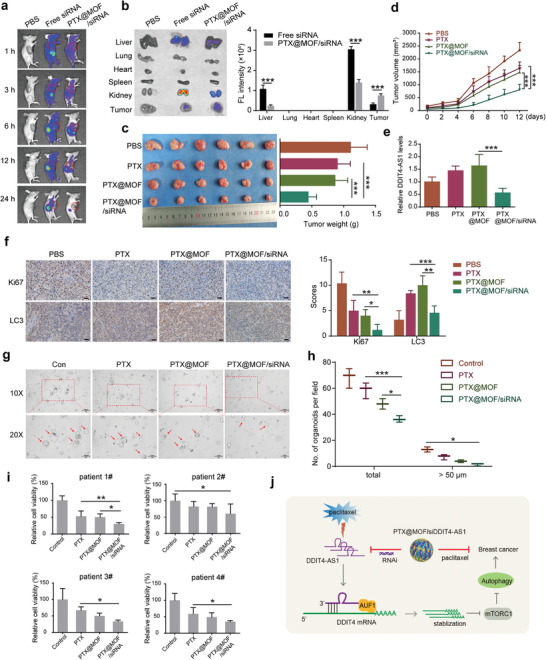
In vivo biodistribution and antitumor activity of the codelivery nanoparticles. a) In vivo fluorescence images of mice were taken at indicated time points postinjection. b) Representative ex vivo fluorescence images and quantitative fluorescence intensity of tumors and organs collected from the mice at 24 h postinjection (*n* = 3 mice per group, ***p < 0.001). c) Image of collected tumors and tumor weight of the MDA‐MB‐231 tumor‐bearing mice treated with the formulas. ***p < 0.001. d) Tumor growth curves of mice after different treatments. ***p < 0.001. e) The quantified DDIT4‐AS1 levels after different treatments by qRT‐PCR analysis. ***p < 0.001. f) Representative Ki67 and LC3 staining and quantitative analysis of orthotopic xenograft sections from different groups. Scale bar, 200 µm. *p < 0.05, **p < 0.01, ***p < 0.001. g,h) Bright‐field images depicting patient‐derived organoid with indicated treatments and number of organoids per field were calculated. *p < 0.05, ***p < 0.001. i) Cell viability was detected using different breast cancer patient‐derived organoids. *p < 0.05, **p < 0.01. j) Schematic of the proposed mechanism of DDIT4‐AS1 in TNBC. Mechanistically, cytosolically localized DDIT4‐AS1 could stabilize DDIT4 mRNA via recruiting the RNA binding protein AUF1 and promoting the interaction between DDIT4 mRNA and AUF1, which subsequently inactivates mTORC1, activates autophagy and promotes breast cancer progression. Besides, the treatment of chemotherapeutic agent paclitaxel (PTX) could induce upregulation of DDIT4‐AS1 and further lead to cyto‐protective autophagy in TNBC, limiting the tumor killing effect. Therefore, a smart core–shell metal–organic framework (MOF) nanosystem was fabricated to effectively load DDIT4‐AS1 siRNA and PTX, providing opportunities for combined gene‐drug TNBC therapy.

Next, we tested our codelivery system in a mouse tumor xenograft model. To determine the accumulation of the nanoparticles in tumors and other major tissues following systemic administration, the siRNA was labeled with cy5.5 fluorescence, and the bio‐distribution of the nanoparticles in the nude mice bearing MDA‐MB‐231 tumors was monitored using an IVIS Lumina III Imaging System. As shown in **Figure** **9**a, free siDDIT4‐AS1 was quickly distributed with no targetability, while the PTX@MOF/siDDIT4‐AS1 nanoparticles displayed an evident signal over time in the tumor area, implying passive tumor accumulations of the NPs. Ex vivo imaging of organs (Figure 9b) showed a stronger fluorescence signal in the tumor of PTX@MOF/siDDIT4‐AS1 group than that of the free siRNA group. In addition, as compared with free siRNA, the nanoparticles accumulated less in the liver and kidney (Figure 9b). These results indicated the long cycling and passive targeting properties of nanoparticles.

The antitumor therapeutic efficacy of the nanoparticles was further evaluated in the TNBC tumor‐bearing mice. Compared with PTX treatment, the antitumor activity was slightly augmented by PTX@MOF likely due to the passive accumulation of the drug in the tumor. Notably, the nanoparticles containing both PTX and DDIT4‐AS1 siRNA achieved a strong tumor‐inhibitory efficacy (Figure 9c,d). Consistent with our previous results, DDIT4‐AS1 was elevated upon treatments of PTX or PTX@MOF, while PTX@MOF/siDDIT4‐AS1 could effectively rescue the level of DDIT4‐AS1 (Figure 9e). Meanwhile, the immunohistochemistry (IHC) staining of tumor slides also indicates that the PTX@MOF/siDDIT4‐AS1 treatment is the most effective in inhibiting tumor growth, as demonstrated by the expressions of Ki67 and LC3 (Figure 9f). The body weight of mice remained unchanged during the treatment period (Figure S8a, Supporting Information), and H&E images of major organs including heart, liver, spleen, lung, and kidney showed no obvious damage after treatment (Figure S8b, Supporting Information). We also measured the blood biochemical indices of liver (ALT and AST) and kidney (CREA2 and UREAL), and these indices were all at normal levels (Figure S8c,d, Supporting Information), indicating no liver or renal toxicity. We evaluated the effect of PTX@MOF/siDDIT4‐AS1 on breast cancer patient‐derived organoids, which more accurately reflect tumor heterogeneity and are emerging as promising preclinical models to predict drug response. Figure 9g–i shows that the PTX@MOF/siDDIT4‐AS1 nanoparticles greatly reduced the quantity and size (>50 µm) of organoids, and the cell viability of four established organoids. These results demonstrated the biocompatibility as well as strong anti‐tumor activity of the nanoparticles.

## 3 Discussion

Autophagy is a highly conserved self‐degradative process that plays a key role in cellular stress responses and survival. Many cancers, including TNBC, become dependent on autophagy as a source of nutrients during tumor growth.^[^ ^34^ ^]^ In the present study, we first revealed that DDIT4‐AS1 is involved in the regulation of the autophagy in TNBC, and knockdown of DDIT4‐AS1 suppressed autophagy activity of TNBC cells. We further demonstrated that silencing of DDIT4‐AS1 significantly inhibited the tumor progression of TNBC in vitro and in vivo. The biological function of DDIT4‐AS1 has been rarely revealed before. It promotes Meningitic *E. coli*‐induced neuroinflammatory responses,^[^ ^17^ ^]^ and is involved in stemness and chemosensitivity of pancreatic cancer.^[^ ^35^ ^]^ Our study is the first to demonstrate a novel role for DDIT4‐AS1 in regulating autophagy, and also reveals its cancer‐promoting role in TNBC. Since autophagy often acts as a pro‐survival response to chemotherapeutic treatment in cancer cells, and suppression of autophagy during chemotherapy has been proposed as a novel therapeutic strategy.^[^ ^34^ ^]^ Here, we demonstrated that paclitaxel treatment increased the expression of DDIT4‐AS1, and silencing DDIT4‐AS1 inhibited autophagy, thereby sensitizing TNBC cells to paclitaxel, suggesting that inhibition of DDIT4‐AS1 may be a potential therapeutic strategy to enhance the efficacy of paclitaxel for TNBC patients.

Compared to traditional therapeutic modalities (such as small molecules and antibodies), oligonucleotides have several advantages for targeting lncRNAs.^[^ ^36^ ^]^ RNA interference (RNAi) technology is the most convenient approach to interfere with disease‐causing or ‐promoting genes, including those that encode “undruggable” proteins. However, due to the polyanionic and biomacromolecular characteristics of siRNAs, specific delivery vehicles are required to facilitate in vivo siRNA delivery. Currently, nanotechnology‐based delivery systems are the most promising tool to deliver siRNA‐based products to cancer cells.^[^ ^37^ ^]^ We have developed a smart core–shell metal–organic framework (MOF) nanosystem for combination of gene therapy with chemotherapeutics, providing an efficacious strategy for enhanced tumor therapy.^[^ ^14^, ^15^, ^33^ ^]^ In this work, considering our research stating that suppression DDIT4‐AS1 sensitizes TNBC to paclitaxel therapy, we take advantage of the biocompatible MOF nanosystem to effectively load DDIT4‐AS1 siRNA and paclitaxel, and investigated its clinical translation potential for effective cancer chemotherapy. The core–shell nanostructure displayed high drug loading efficiency and colloidal stability. At cellular level, the nanoparticles acted as an effectively transfection agent to facilitate the intracellular delivery and endo/lysosome escape of the payloads. Upon intravenous injection, the nanoparticles could passively accumulate into tumor via the well‐defined EPR effect, and impose robust potent gene‐silencing efficacy, and thus sensitize chemotherapy to inhibit tumor growth. The new RNAi platform developed in this work provides an efficient and safe approach to co‐delivery of siRNA and paclitaxel via core–shell delivery nanosystem, providing opportunities for combined gene‐drug TNBC therapy.

We found that DDIT4‐AS1 was highly expressed in TNBC cell lines and clinical breast cancer samples, indicating that DDIT4‐AS1 possess potential to be a biomarker for the clinical diagnosis and treatment of TNBC. A study has reported that the overexpressed DDIT4‐AS1 in PDAC was regulated by ALKBH5 in an m^6^A‑dependent manner, and recruitment of HuR onto m^6^A‐modifed sites is essential for DDIT4‐AS1 stabilization.^[^ ^35^ ^]^ Apart from this, to figure out the reason for the upregulated DDIT4‐AS1 in TNBC, we analyzed the promoter region of DDIT4‐AS1 by genome bioinformatics analysis (http://genome.ucsc.edu/) and identified that H3K27ac was highly enriched in this region. Recent progress in epigenetics and chromatin biology indicate that active promoters carry unique epigenetic marks, including acetylation of various residues of histones H3 and H4 (H3K27ac for instance) and H3K4me3,^[^ ^38^ ^]^ which subsequently accounting for the aberrant expression of lncRNAs. For instance, the histone deacetylase HDAC2 could inhibit lncRNA H19 expression by histone H3K27 deacetylation in its promoter via binding with SP1.^[^ ^39^ ^]^ Oct4, a key stemness transcription factor, transcriptionally activates lncRNA NEAT1 via promoter and lncRNA MALAT1 via enhancer binding to promote cell proliferation and motility, and led to lung tumorigenesis and poor prognosis.^[^ ^40^ ^]^ Here, we found high enrichment of H3K27ac at the promoter of DDIT4‐AS1 in TNBC cell lines and tumor tissues, and the H3K27ac of DDIT4‐AS1 is positively associated with its expression. These data confirm that DDIT4‐AS1 is frequently increased in breast cancer, and histone acetylation activation of promoter may partly account for this dysregulation. Collectively, our study innovatively revealed the epigenetic regulation of DDIT4‐AS1 from the perspective of histone acetylation modification, and enriched the in‐depth research of lncRNA DDIT4‐AS1.

Precedent genome‐wide studies reported that antisense lncRNAs regulated the proximal target mRNA expression, such as Xist,^[^ ^41^ ^]^ NAMPT‐AS,^[^ ^9^ ^]^ and EGOT.^[^ ^22^ ^]^ UCSC genome browser shows that DDIT4‐AS1 localizes in proximity to the DDIT4 transcription start site and is transcribed in an opposite direction from DDIT4. We further demonstrated that DDIT4‐AS1 positively regulate the levels of mRNA and protein of its proximal gene DDIT4. DNA damage‐inducible transcript 4 (DDIT4), variously termed REDD1 or RTP801, is induced by a variety of stress conditions, including oxidative stress, endoplasmic reticulum stress, hypoxia, and starvation.^[^ ^42^ ^]^ Over the past decades, DDIT4 dysregulation has been observed in numerous human malignancies, such as prostate cancer, ovarian cancer, gastric cancer, and breast cancer.^[^ ^24^, ^42^ ^]^ Moreover, DDIT4 inhibits mammalian target of rapamycin complex 1 (mTORC1) by stabilizing the tuberous sclerosis complex (TSC1–TSC2). DDIT4‐mediated mTOR repression produces the lack of phosphorylation of the ULK complex, turning the complex in a closed structural conformation (active form) and triggering the formation of the autophagosome.^[^ ^43^ ^]^ Consistently, we demonstrated that DDIT4‐AS1 induced autophagy largely dependent on DDIT4‐mediated the inactivation of mTOR signaling pathway. We further explored the mechanism underlying the regulation of DDIT4 mRNA by DDIT4‐AS1, and found that DDIT4‐AS1 mainly localizes in cytoplasm and maintains stability of DDIT4 mRNA. Posttranscriptional gene regulation by lncRNAs is always mediated by associating with RNA‐binding proteins (RBPs), which are known to modulate the expression, stability, maturation, and transport of target mRNAs.^[^ ^27^ ^]^ For example, lncRNA HMS functions as a HOXC10 mRNA stabilizing factor by associating with the HuR to stabilize HOXC10 mRNA, which has an essential role in the proliferation of cancer cells.^[^ ^44^ ^]^ In our study, we found that DDIT4‐AS1 could directly bind to DDIT4 mRNA. Furthermore, DDIT4‐AS1 was also shown to interact with AUF1, one of the best‐characterized AU‐rich elements (ARE)‐binding protein,^[^ ^45^ ^]^ by its 5’ region to enhance the interplay between 3’UTR of DDIT4 mRNA and AUF1, thereby stabilizing and protecting the degradation of DDIT4 mRNA. Consistent with our results, DDIT4‐AS1 promoted the stability of DDIT4 mRNA in the progress of *E. coli* infection.^[^ ^17^ ^]^ However, contrary to our results, the overexpressed DDIT4‐AS1 in PDAC recruits UPF1 to destabilize DDIT4 mRNA.^[^ ^35^ ^]^ We consider that the regulation of DDIT4 by DDIT4‐AS1 may be inconsistent due to the different types of pathologic conditions and the different intermediate regulatory proteins.

In summary, our study is the first to illustrate the importance of lncRNA DDIT4‐AS1 in breast tumorigenesis, as shown in the working model (Figure 9j). We found that knockdown of DDIT4‐AS1 blocked the activation of autophagy, thereby inhibiting breast cancer cell growth and migration. A smart nanosystem assembled by a pure paclitaxel core and a siDDIT4‐AS1‐encapsulating MOF shell was successfully constructed for drug–gene combinations, and exerted significant antitumor activity against TNBC in vitro, in vivo and in the organoids derived from patient samples. Our findings suggest that the “DDIT4‐AS1/DDIT4/autophagy” pathway may be further explored as a novel target for developing therapeutic strategies to treatment of TNBC, and codelivery of DDIT4‐AS1 siRNA and paclitaxel via core–shell delivery nanosystem may be a promising synergistic strategy.

## 4 Experimental Section

### RNA Sequencing

Cell RNA‐seq (3 replicates each group) was done in Ribobio (Ribobio, Guangzhou, China). Briefly, total RNA was extracted using TRIzol (Biomade), rRNA was removed using a ribosomal RNA depletion kit, and intact RNA was fragmented, end repaired, adapter ligated, and PCR amplified following the Illumina protocol. Libraries were sequenced by IlluminaHiSeq 3000 platform at Guangzhou RiboBio Co., Ltd. (Guangzhou, China).

### Breast Specimens and Clinical Assessments

In total, 69 pair of breast cancer tissues and normal tissues were obtained from The Second Xiangya Hospital, Central South University (Changsha, China). All samples were frozen in liquid nitrogen immediately after surgical resection. This study conformed to the clinical research guidelines and was approved by the research ethics committee of The Second Xiangya Hospital. Written informed consent from all patients were obtained.

### Patient‐Derived Organoid

The generation of patient‐derived organoid and drug response assay were performed as described in a previous study.^[^ ^16^ ^]^ Briefly, breast cancer tissue was cut into 1–3 mm^3^ pieces and was digested in collagenase (Sigma). Dissociated cell clusters were resuspended in 50% cold Matrigel (Corning) and seeded in a prewarmed 6‐well plate (Corning) at 25 µL drops. The drops were solidified in a 37 °C and 5% CO_2_ incubator for 30 min, and then 2.5 mL organoid culture medium was added to each well and refreshed every 2–3 days. Organoids were dissociated into smaller clusters and resuspended in 2.5% Matrigel/modified culture medium. Approximately 2000 cells in 54 µL were seeded in each well of the type‐I collagen gel precoated 384‐well plate. Forty‐eight hours after seeding, 6 µL of a tenfold dilution series of each compound was dispensed, and at least three technical replicates of each drug were tested. After 4 days of drug incubation, cell viability was detected using the CellTiter‐Glo 2.0 assay (Promega) according to the manufacturer's instructions.

### Reagents and Antibodies

Paclitaxel (NSC 125 973), cisplatin (s1166), and doxorubicin (s1208) were purchased from Selleck. Actinomycin D (HY‐17559) and rapamycin (HY‐10219) were purchased from MCE. CQ (C6628) was purchased from Sigma. Antibodies used in immunoblotting: mTOR (2972S, 1:1000), p‐mTOR (Ser2448) (5536S, 1:1000), SQSTM1/p62 (88588S, 1:1000), BECN1 (D40C5, 1:1000), LC3 (3868S, 1:1000) were purchased from Cell Signaling Technologies. Anti‐REDD1 (10638‐1‐AP, 1:1000), anti‐AUF1 (12770‐1‐AP, 1:1000), anti‐ZFP36 (12737‐1‐AP, 1:1000), anti‐KHSRP (55409‐1‐AP, 1:1000), anti‐*β*‐actin (20536‐1‐AP,1:5000), were purchased from Proteintech. GAPDH (GB11002) was purchased from servicebio. LC3 (L7543, 1:2000) was purchased from Sigma. Ki67 (MAB‐0672) was purchased from MXB Biotechnologies. Anti‐H3K27 (ab4729, abcam), anti‐TCF4 (22337‐1‐AP, proteintech) and normal IgG (2729, CST) were used in the CHIP assay. Normal IgG/Peroxidase‐conjugated AffiniPure Goat Anti‐Rabbit/Mouse IgG (H+L) was purchased from Jackson Immuno Research.

### Cell Lines and Culture

The human breast cancer cell lines, BT549 and HCC1806, were cultured in RPMI‐1640 medium, MDA‐MB‐231, MCF‐7, T47D, and MDA‐MB‐436 were cultured in Dulbecco's modified Eagle's medium. MCF10A cells were cultured in Clonetics MEGM mammary epithelial cells growth medium (Lonza Walkersville, Inc. Walkersville, MD) supplemented with 5% horse serum (Sigma‐Aldrich, St. Louis, MO), hEGF (Sigma‐Aldrich, 20 ng mL^−1^), hydrocortisone (Sigma‐Aldrich, 0.5 µg mL^−1^), cholera toxin (Sigma‐Aldrich, 1 ng mL^−1^), and insulin (Sigma‐Aldrich, 10 µg mL^−1^). All cell culture media were supplemented with 10% fetal bovine serum, 100 units mL^−1^ penicillin and 100 µg mL^−1^ streptomycin. All cell lines were maintained at 37 °C in a humidified atmosphere containing 5% CO_2_/95% air. Cell lines were authenticated using STR profile analysis and used within 3–20 passages of thawing the original stocks.

### RNA Extraction, Reverse Transcription, and Quantitative RT‐PCR

Total RNA was isolated using TRIzol reagent (Biotech, USA). Complementary DNA (cDNA) was synthesized using a PrimeScript RT reagent kit (TaKaRa, Japan). Real‐time PCR was performed using QuantStudio Design & Analysis Software v1.5.1 on a QuantStudio Real‐Time PCR Instrument (life technologies, USA). Relative RNA abundances were calculated by the standard 2^−ΔΔCt^ method.

### siRNA, shRNA, and Plasmid Transfection

siRNAs were purchased from Ribobio or Qingke. Transfection of siRNA was carried out according to the manufacturer's protocol. Briefly, cells in exponential phase of growth were plated in six‐well tissue culture plates at 1 × 10^5^ cells per well, grown for 24 h, and then transfected with siRNA using lipofectamine RNAimax reagent and OPTI‐MEM I‐reduced serum medium. To stably silence DDIT4‐AS1 expression, the DDIT4‐AS1‐targeted shRNA lentiviral particles (GENE) were transduced into cells, and the cells stably expressing the shRNA were then selected with 2 µg mL^−1^ of puromycin for 7 days. Transfection of the plasmid was carried out using lipofectamine 2000 (Invitrogen) reagent according to the manufacturer's protocol. The si and shRNA sequences were showed in Table S5 (Supporting Information).

### Western Blot Assay

Cells were lysed at ice for 30 min in RIPA supplemented with a protease inhibitor cocktail (Biotool), followed by centrifugation at 12 000 rpm for 15 min. Proteins (20–40 µg) were resolved by SDS‐PAGE and then transferred to PVDF membrane. The PVDF membranes were incubated with the respective antibodies in 5% BSA at 4 °C overnight, followed by incubation with a secondary antibody at room temperature for 1 h. The protein signals were detected by ECL method.

### Clonogenic Assay

Cells were plated in 6‐well tissue culture plates (800 cells per well) and incubated at 37 °C in a humidified atmosphere containing 5% CO_2_/95% air for 15 days. At the end of incubation, cells were fixed with 4% paraformaldehyde and stained with crystal violet for 20 min, washed with PBS, and then the colonies were counted.

### 5‐Ethynyl‐2’‐deoxyuridine Assay

The cells were incubated with 5‐Ethynyl‐2’‐deoxyuridine assay (EdU; Ribobio) for 2 h, and processed according to the manufacturer's instruction. After three washes with PBS, the cells were incubated with 100 µL of 1 × Apollo reaction cocktail for 30 min. Then cells were washed three times with 0.5% Triton X‐100. The DNA contents were stained with 100 µL of 1 × Hoechst 33 342 (5 µg mL^−1^) for 30 min and visualized under a fluorescence microscope.

### Subcellular Fractionation

Nuclear/cytoplasmic isolation was carried out by using the PARIS kit (Am1921, Thermo Fisher Scientific, USA) according to the manufacturer's protocol. Subcellular fractions were prepared as follows. Cytoplasmic and nuclear fractions were divided for RNA extraction. GAPDH and U6 were used as qRT‐PCR markers of cytoplasmic and nuclear RNAs, respectively.

### Transmission Electron Microscopy

Cells were fixed with 2.5% glutaraldehyde and stored at 4 °C until embedding. The samples were postfixed with 1% osmium tetroxide. and then dehydrated by using graded acetone. Specimens were embedded and cut into ultrathin section (50–100 nm). Sections were stained with 3% uranyl acetate and lead citrate. Images were examined with a HT7700 transmission electron microscope (Hitachi, Japan).

### Fluorescence In Situ Hybridization (FISH) Assay

The FISH assay was performed in MDA‐MB‐231 cells according to the specifications of the manufacturers. The Cy3‐labeled lncRNA DDIT4‐AS1 probes used in the study were designed and synthesized by Ribobio (Guangzhou, China). Briefly, the prepared cells were fixed with 4% paraformaldehyde for 30 min. After permeabilization, the cells were incubated with specific probes at 37 °C overnight. The cell nuclei were stained with DAPI (Sigma‐Aldrich, USA). The staining results were observed using a confocal microscope.

### Tandem mRFP‐GFP Fluorescence Microscopy

To evaluate tandem fluorescent LC3 puncta, 48 h after mCherry‐GFP‐LC3 transfection, cells were washed once with 1 × PBS, incubated with EBSS (Hyclone) for the indicated durations. and then the cells were fixed and sent out for confocal microscopy analysis.

### RNA Pull‐Down Assay

RNAs were in vitro transcribed using MAXIscript T7 In Vitro Transcript Kit (thermo AM1312). Transcribed RNAs were biotin labeled with Pierce RNA 3’End Desthiobiotinylation Kit (thermo 20163). Positive, negative, and biotinylated RNAs were mixed and incubated with MDA‐MB‐231 cell lysates. Then RNA pull down assay was carried out by using a Pierce Magnetic RNA‐protein Pull Down Kit according to the manufacturer's instructions (thermo 20164). Beads were washed with washing buffer. For protein analysis, the beads were boiled in 1 × loading buffer for 10 min, while the RNA present in the complexes was isolated and analyzed by qRT‐PCR.

### RIP Assay

RIP was implemented using a Magna RIPRNA‐Binding Protein Immunoprecipitation Kit (Millipore, Cambridge, MA) as directed by the manufacturer. Post the harvest of cells in IP lysis buffer and mechanical shear by a homogenizer, antibodies against AUF1 (ab259895, abcam) and IgG (2729, CST) were added and cultured with the cell extract overnight under 4 °C. Then streptavidin‐coated magnetic beads were added for incubation for 2 h. The isolated and purified RNAs were subjected to qRT‐PCR measurement.

### Chromatin Immunoprecipitation (ChIP)

The chromatin or DNA‐protein complex was isolated according to the manufacturer's instruction (Abcam, ab117152‐Chromatin Extraction Kit). Then Chromatin immunoprecipitation (CHIP) experiment was carried out by using a ChIP assay kit according to the manufacturer's instructions (Abcam, ab117138‐ChIP Kit‐One Step). Quantitative real‐time polymerase chain reaction (qPCR) was performed to measure the ChIP signal, and enrichment of target was analyzed based on input DNA and normal IgG signals. The following specific primers were used in the CHIP‐qPCR analysis: DDIT4‐AS1 promoter (5’‐CTGTCTGGGCCTTCTAACCG‐3’ and 5’‐ CTGTGCTGGCTGAAGCTACT ‐3’).

### Preparation of PTX@MOF/siDDIT4‐AS1

Paclitaxel (PTX) NPs were prepared by quickly adding 50 µL of a PTX solution (10 mg mL^−1^, dissolved DMSO) in 5 mL of ultrapure water under vigorous stirring, followed by sonication for 30 s. Then, 50 µL of TA (40 mg mL^−1^) and 50 µL of FeCl_3_ (10 mg mL^−1^) were added to the above NPs, followed by sonication for 2 min and fully vortex. Then, 62.5 µL of siDDIT4‐AS1 (20 µm) was added and incubated for 30 min. Finally, the PTX@MOF/siDDIT4‐AS1 was collected by centrifugation and washed with ultrapure water. In the visualization of experiments in vitro and in vivo, FAM or cy5.5 labeled siDDIT4‐AS1 was used to form nanoparticles.

### Characterization of PTX@MOF/siDDIT4‐AS1

The particle size and the *ζ* potential of PTX NPs and PTX@MOF/siDDIT4‐AS1 NPs were determined by dynamic light scattering (DLS) analysis using a Malvern Zetasizer Nano series (Nano ZS, Malvern instruments). The morphology of PTX@MOF/siDDIT4‐AS1 NPs were observed using transmission electron microscopy–energy‐dispersive spectrometry (TEM−EDS, Titan G2 60‐300, FEI). The entrapment efficiency (EE%) and loading capacity (LC%) of PTX were determined using high‐performance liquid chromatography (HPLC, Agilent 1260 Infinity II).

To measure the siDDIT4‐AS1 loading capacity, different concentrations (1, 2, 4 µM) of siDDIT4‐AS1 was used to prepare PTX@MOF/siDDIT4‐AS1, and the unloaded siRNA was collected via centrifugation at 20 000 rpm for 30 min, followed by quantification using polyacrylamide gel electrophoresis (PAGE). The polyacrylamide gel stock solution containing 4.8 g of urea, 5 mL of acrylamide (30%), and 1 mL of 10 × TBE buffer was mixed with 32 µL of APS and 5 µL of TEMED to prepare a solid gel. The electrophoresis experiment was carried out at a constant potential of 100 V in 1 × TBE buffer for 30 min, and the images of the gel were acquired for analysis by a gel imaging system (FluorChem Imaging System, Protein‐Simple) under 365 nm UV irradiation.

### Cellular Uptake Assay

MDA‐MB‐231 and BT549 cells were seeded in a 35 mm glass‐bottom Petri dish. After incubation with PTX@MOF/siDDIT4‐AS1‐FAM for 4 h, the cells were washed twice with PBS. Then, 500 µL of the Lyso‐Tracker Red working solution (80 nm) was added to the stained endo/lysosome for 30 min, and nuclei were labeled with Hoechst 33 342 (1 µg mL^−1^) for 15 min. The cells were imaged using a fluorescence microscope (OLYMPUS IX73, Japan).

### In Vivo/Ex Vivo Fluorescence Imaging

The animal protocol was in accordance with the institutional guidelines of the Animal Care and Use Committee of Central South University. MDA‐MB‐231 cells suspended in 100 µL of PBS were subcutaneously injected into the armpit of each female BALB/c nude mouse (6 week old), and the mice were treated with different formulations when the volume of tumors grew to ≈100 mm^3^. For a biodistribution study, the mice were intravenously administrated free cy5.5‐siDDIT4‐AS1 or PTX@MOF/cy5.5‐siDDIT4‐AS1 and imaged using a PerkinElmer in vivo optical imaging system (IVIS Lumina) at indicated time points postinjection. The mice were sacrificed 24 h after injection to obtain the major organs (heart, liver, spleen, lung, and kidney) and tumors for ex vivo imaging. The images were analyzed with Living Imaging Software.

### In Vivo Antitumor Study

To investigate whether DDIT4‐AS1 could attenuate chemosensitivity of breast cancer cells to paclitaxel in vivo, MDA‐MB‐231 cells with stable expression of a DDIT4‐AS1‐targeted shRNA or a nontargeting shRNA were subcutaneously injected into nude mice. The mice were randomly divided into four groups: shNT+vehicle, shNT+PTX, shDDIT4‐AS1+vehicle, shDDIT4‐AS1+PTX, and 10 mg kg^−1^ PTX was given intraperitoneally once every 3 days, and tumor volumes were measured on the days as indicated. To evaluate antitumor effects of nanoparticles, the MDA‐MB‐231 tumor‐bearing nude mice were randomly assigned into four groups and intravenously injected at day 0, 3, 6, and 9 with the following formulations: PBS, PTX, PTX@MOF, and PTX@MOF/siDDIT4‐AS1 (200 µL, 10 mg kg^−1^ PTX, 1 nmol siRNA dose per mouse). Subsequently, the tumor volume and body weight of each mouse were monitored every other day for 2 weeks. At the termination of the experiment, mice were sacrificed and major organs and tumors were collected. The photographs of tumors were taken using a high‐quality camera. Then, major organs (heart, liver, spleen, lung, and kidney) and tumors were immersed in 4% formaldehyde, followed by embedding and slicing. Finally, the slides were stained with hematoxylin & eosin (H&E) and observed by an optical microscope.

### Biochemical Analysis

BALB/c mice were randomly divided into four groups (*n* = 4) for indicated treatments. Then, the mice blood was harvested from the eyeballs and serums were obtained through centrifugation at 3000 rpm for 10 min and stored at −80 °C for biochemical analyses to detect toxicity of the liver (ALT, AST) and the kidney (BUN, CRE).

### Statistical Analysis

Two group comparisons were analyzed using the Student's *t*‐test. Comparison of multiple groups (> 2) were conducted using two‐way ANOVA. Results are shown as the mean ± s.d. of multiple independent experiments. Graphpad Prism 6.0 was used to perform statistical analysis. *p* < 0.05 was considered statistically significant. All experiments were performed at least three times.

### Ethical Statement

Animal studies were approved by the Ethics Committee of The Second Xiangya Hospital, and the animal protocol was in accordance with the institutional guidelines of the Animal Care and Use Committee of Central South University. All participants have given informed consent before scientific research.

## Conflict of Interest

The authors declare no conflict of interest.

## Author Contributions

Y.C. and W.H.Z. designed, conceived the study, and revised the manuscript. T.J., J.J.Z., R.G., C.X.Z., and Y.Y.C. performed the experiments and analyzed data. T.J. drafted the manuscript. J.M.Y., W.J.Y., Z.L.C., and S.L.J. provided technological support in the experiments. All authors approved the manuscript for submission and consented for publication.

## Supporting information

Supporting Information

## Data Availability

The data that support the findings of this study are available in the supplementary material of this article.
