# Correction to “Targeting lncRNA DDIT4‐AS1 Sensitizes Triple Negative Breast Cancer to Chemotherapy via Suppressing of Autophagy”

**DOI:** 10.1002/advs.74387

**Published:** 2026-02-17

Ting Jiang, Jiaojiao Zhu, Shilong Jiang, Zonglin Chen, Ping Xu, Rong Gong, Changxin Zhong, Yueying Cheng, Xinyuan Sun, Wenjun Yi, Jinming Yang, Wenhu Zhou, Yan Cheng. “Targeting lncRNA DDIT4‐AS1 Sensitizes Triple Negative Breast Cancer to Chemotherapy via Suppressing of Autophagy.” DOI: https://doi.org/10.1002/advs.202207257. Adv Sci (Weinh) 2023 June; 10(17):2207257

In this article, the authors recently noticed the following figures were inadvertently misplaced: the invasion image (BT549 shNT group, Figure 4g), the migration image (MDA‐MB‐231 Con group, Figure 8f), and the LC3 blot (siATG5 group, Figure S4b). The amended figures are now shown below. The conclusions of this paper are not affected.

Corrected Figure 4g is shown below:


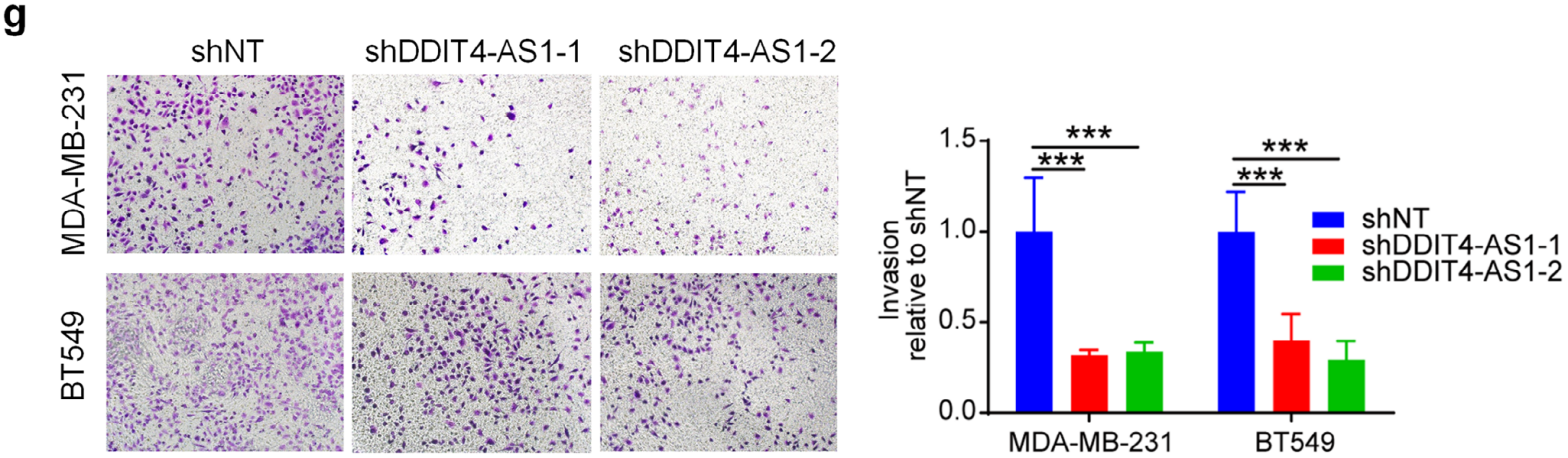


Corrected Figure 8f is shown below:


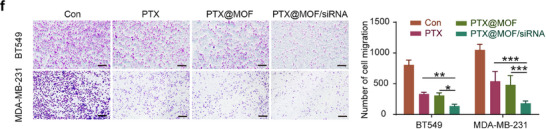


Corrected Figure S4b is shown below:


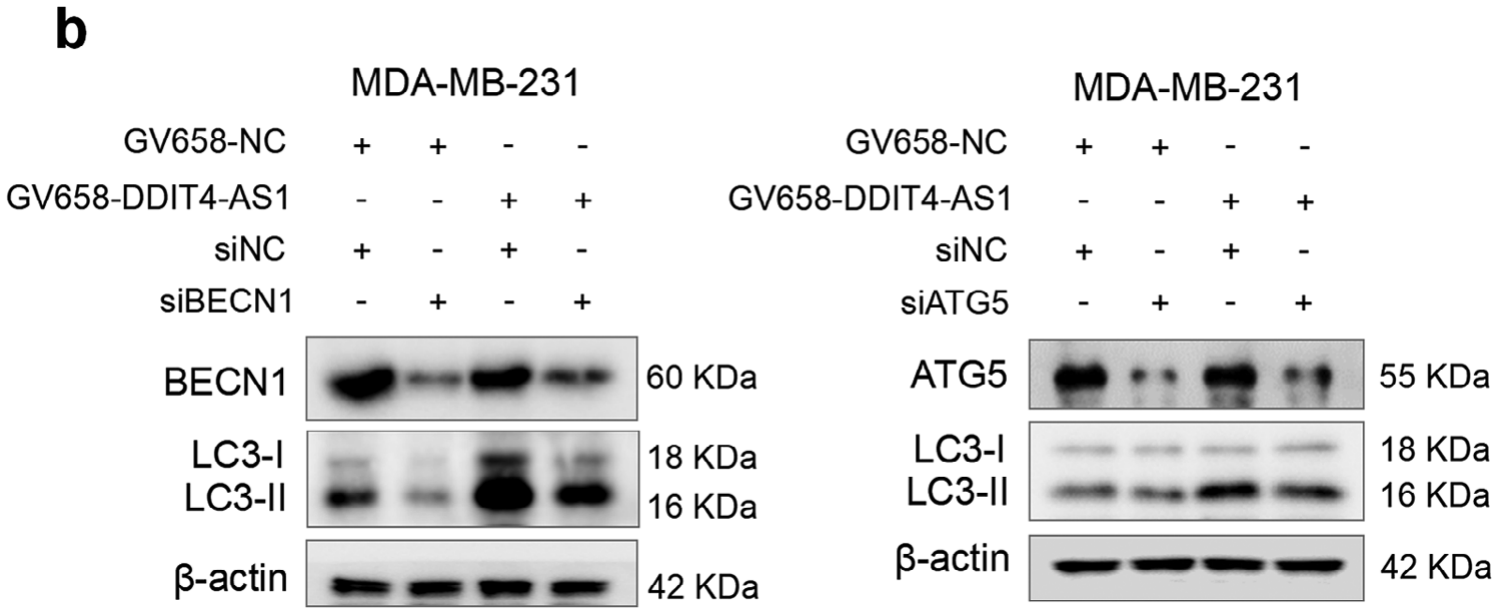


The authors sincerely apologize for these mistakes.

