# Supplementary material for: Targeting lncRNA DDIT4‐AS1 Sensitizes Triple Negative Breast Cancer to Chemotherapy via Suppressing of Autophagy
Source: Adv Sci (Weinh). 2023 Apr 25;10(17):2207257. doi: 10.1002/advs.202207257 (PMC10265098; doi:10.1002/advs.202207257)
Supplement: Supplementary file 1 — Supporting Information [file ADVS-10-2207257-s001.pdf]

ADVANCED SCIENCE
Open Access

Supporting Information

for *Adv. Sci.*, DOI 10.1002/advs.202207257

Targeting lncRNA DDIT4-AS1 Sensitizes Triple Negative Breast Cancer to Chemotherapy via Suppressing of Autophagy

*Ting Jiang, Jiaojiao Zhu, Shilong Jiang, Zonglin Chen, Ping Xu, Rong Gong, Changxin Zhong, Yueying Cheng, Xinyuan Sun, Wenjun Yi, Jinming Yang, Wenhu Zhou and Yan Cheng**

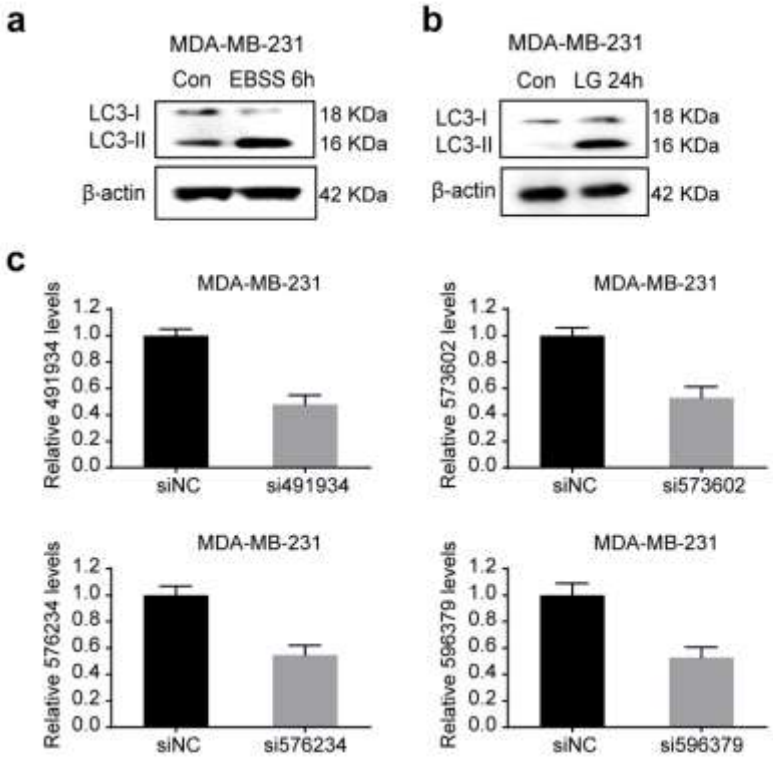

Supplementary Fig. 1 Screening of autophagy related lncRNAs. a Western blot showing the LC3-II/LC3-I ratio of MDA-MB-231 cells was induced by EBSS, and RNA-seq was taken from the same batch of cell samples. **b** Western blot confirming the LC3-II/LC3-I ratio of MDA-MB-231 cells was increased by low glucose medium treatment. **c** qRT-PCR analysis was used to detect the silencing efficacy of siRNAs of four lncRNAs in MDA-MB-231 cells. β-actin was the internal control.

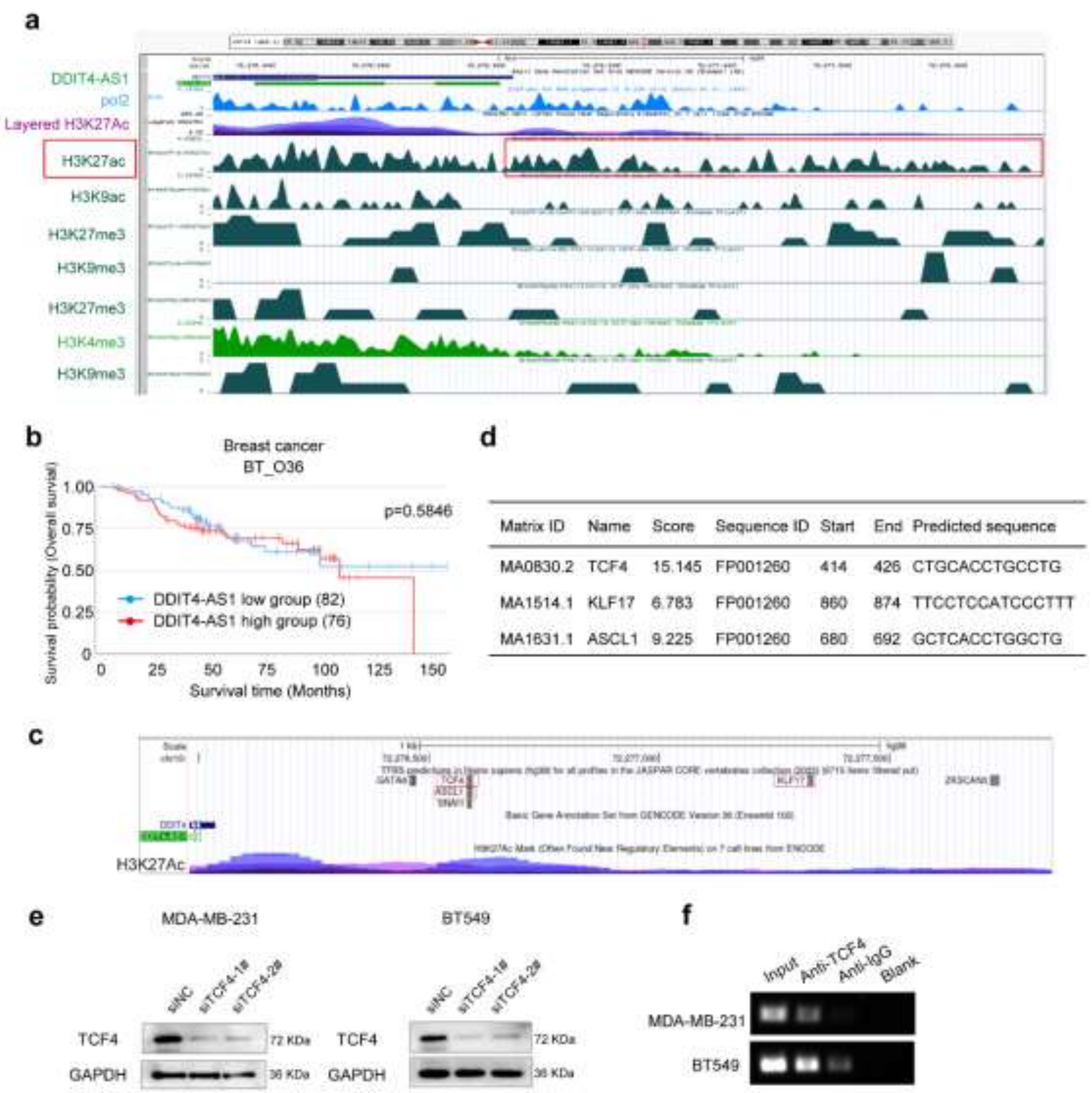

Matrix ID	Name	Score	Sequence ID	Start	End	Predicted sequence
MA0830.2	TCF4	15.145	FP001260	414	426	CTGCACCTGCCTG
MA1514.1	KLF17	6.783	FP001260	860	874	TTCCTCCATCCCTTT
MA1631.1	ASCL1	9.225	FP001260	680	692	GCTCACCTGGCTG

Supplementary Fig. 2 The lncRNA DDIT4-AS1 is activated by H3K27ac and TCF4. a The gene annotation information in UCSC database indicated histone modifications of DDIT4-AS1 promoter. **b** The association between DDIT4-AS1 expression and overall survival of breast cancer patients was obtained from the LnCAR database. **c** Online prediction from JASPAR TFBS in the UCSC Genome Browser suggested that TCF4, ASCL1 and KLF17 are potential transcription factors of DDIT4-AS1. **d** The JASPAR database was used to predict the binding site sequences of the candidate transcription factors in the promoter regions of DDIT4-AS1. **e** MDA-MB-231 and BT549 cells were transfected with the indicated siRNAs, and the expression of TCF4 was examined by western blot. **f** CHIP and RT-PCR assays using anti-TCF4 antibody showed that TCF4 was enriched at the

promoter region of DDIT4-AS1.

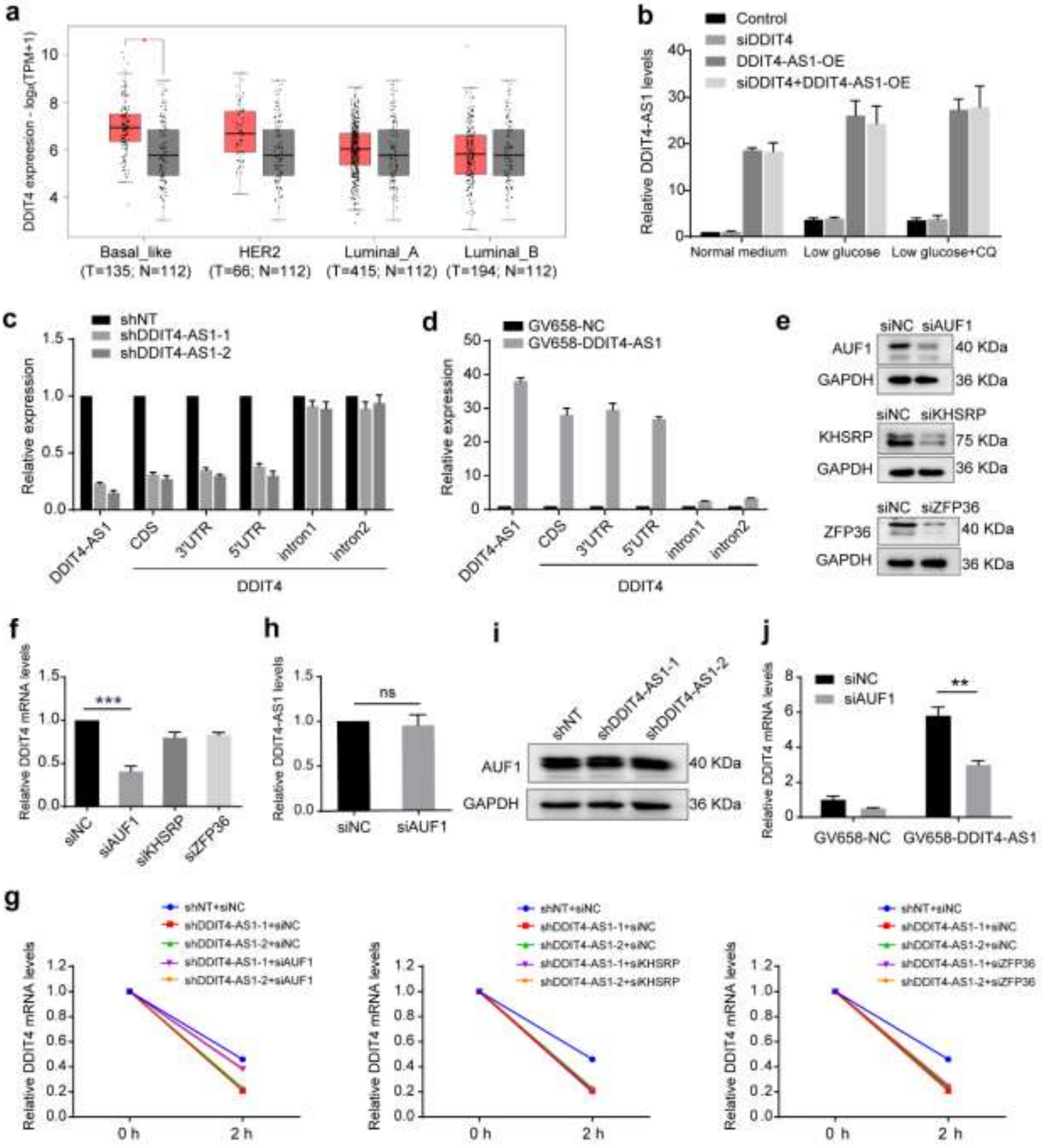

Supplementary Fig. 3 DDIT4-AS1 posttranscriptional regulates DDIT4 via AUF1. a. The expression of DDIT4 among different types of breast tumor and non-tumor tissues were obtained from the GEPIA2.0 database. **b.** qRT-PCR analysis of DDIT4-AS1 levels in MDA-MB-231 cells treated with CQ, different medium condition, or subjected to DDIT4-AS1 overexpression or DDIT4 knockdown. β-actin

was used as the internal control. **c-d** qRT-PCR analysis of DDIT4 pre-mRNA (intron1, intron2) and mature mRNA (3'-UTR, CDS and 5'-UTR) expression in MDA-MB-231 cells with DDIT4-AS1 knockdown (c) or overexpression (d). β-actin was the internal control. **e** Western blot showing the knockdown effects of siRNAs. **f** MDA-MB-231 cells were transfected with indicated siRNAs, qRT-PCR was used to detect the expression of DDIT4. β-actin was the internal control. ***$p < 0.001$. **g** 2.5 μg/ml ActD were used to block mRNA transcription. qRT-PCR was used to detect the effect of DDIT4-AS1 knockdown and siNC/siKHSRP/siZFP36/siAUF1 on the degradation rate of DDIT4 mRNA in MDA-MB-231. 18s was the internal control. **h** qRT-PCR analysis of DDIT4-AS1 expression in MDA-MB-231 cells with AUF1 silencing. β-actin was the internal control. **i** Western blotting was used to detect the effect of DDIT4-AS1 knockdown on the expression of AUF1 protein in MDA-MB-231. **j** QRT-PCR analysis of DDIT4 mRNA levels in MDA-MB-231 cells transfected with siRNAs or vectors as indicated. β-actin was the internal control. **$p < 0.01$.

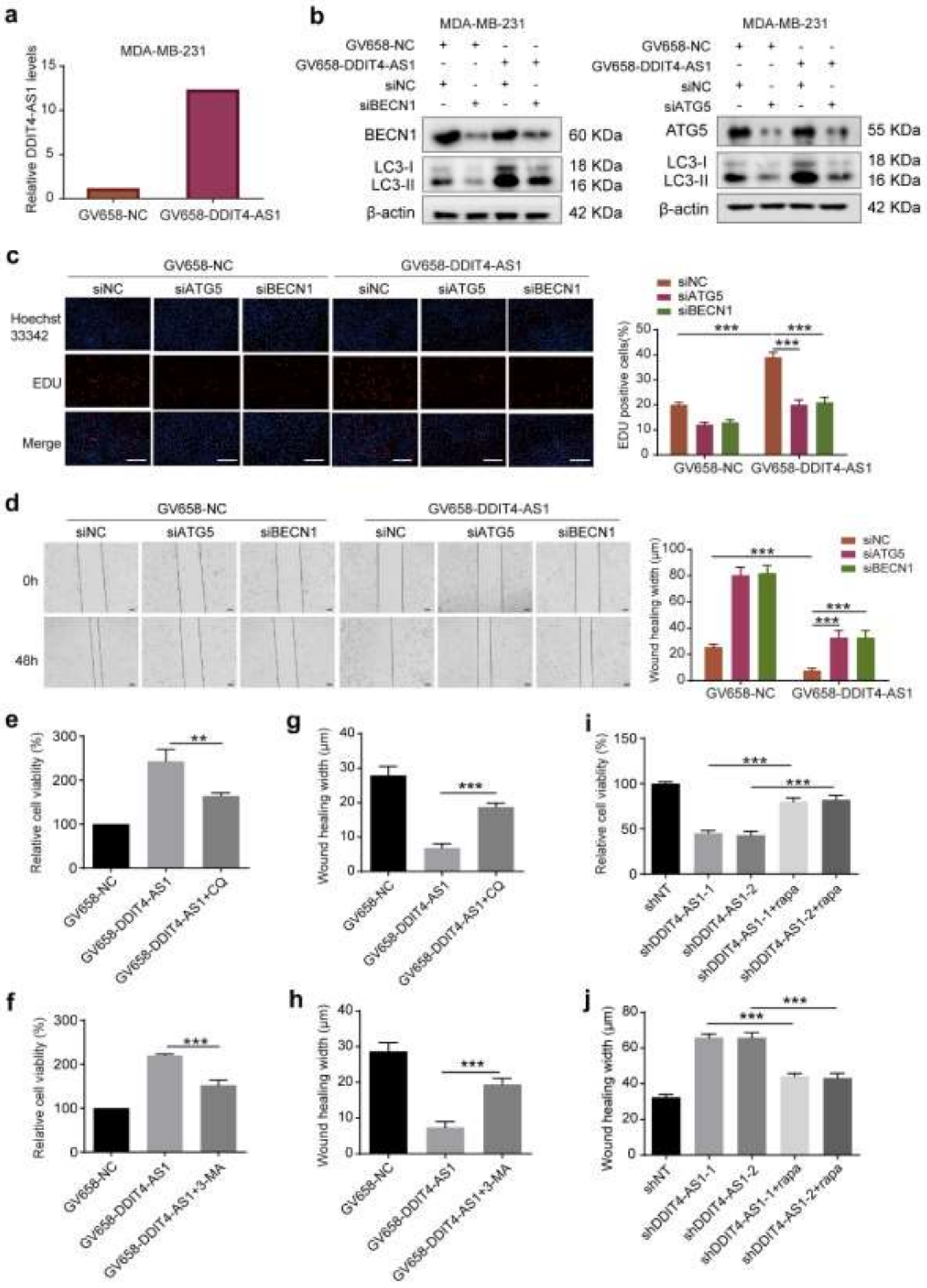

Supplementary Fig. 4 DDIT4-AS1 induces higher autophagy activity of MDA-MB-231 cells. a qRT-PCR was performed to measure the expression level of DDIT4-AS1 in MDA-MB-231 transfected with GV658-NC or GV658-DDIT4-AS1.

β-actin was the internal control. **b** Western blot was used to detect indicated proteins in MDA-MB-231 with various treatments. **c** EDU assay detecting EDU positive cells of MDA-MB-231 subjected to various treatments. Scale bar, 200 μm. ***$p < 0.001$. **d** Scratch assay showing the effect of various treatments on cell migration of MDA-MB-231. Scale bar, 100 μm. ***$p < 0.001$. **e-f** 48 hours after cell seeding, CCK8 assay was used to examine cell viability of MDA-MB-231 cell subjected to various treatments. **$p < 0.01$.***$p < 0.001$. **g-h** 48 hours after cell scratching, wounding healing width was measured to show the effect of various treatments on cell migration of MDA-MB-231. ***$p < 0.001$.**i** 48 hours after cell seeding, CCK8 assay was used to examine cell viability of MDA-MB-231 cell subjected to various treatments. ***$p < 0.001$. **j** 48 hours after cell scratching, wounding healing width was measured to show the effect of various treatments on cell migration of MDA-MB-231.

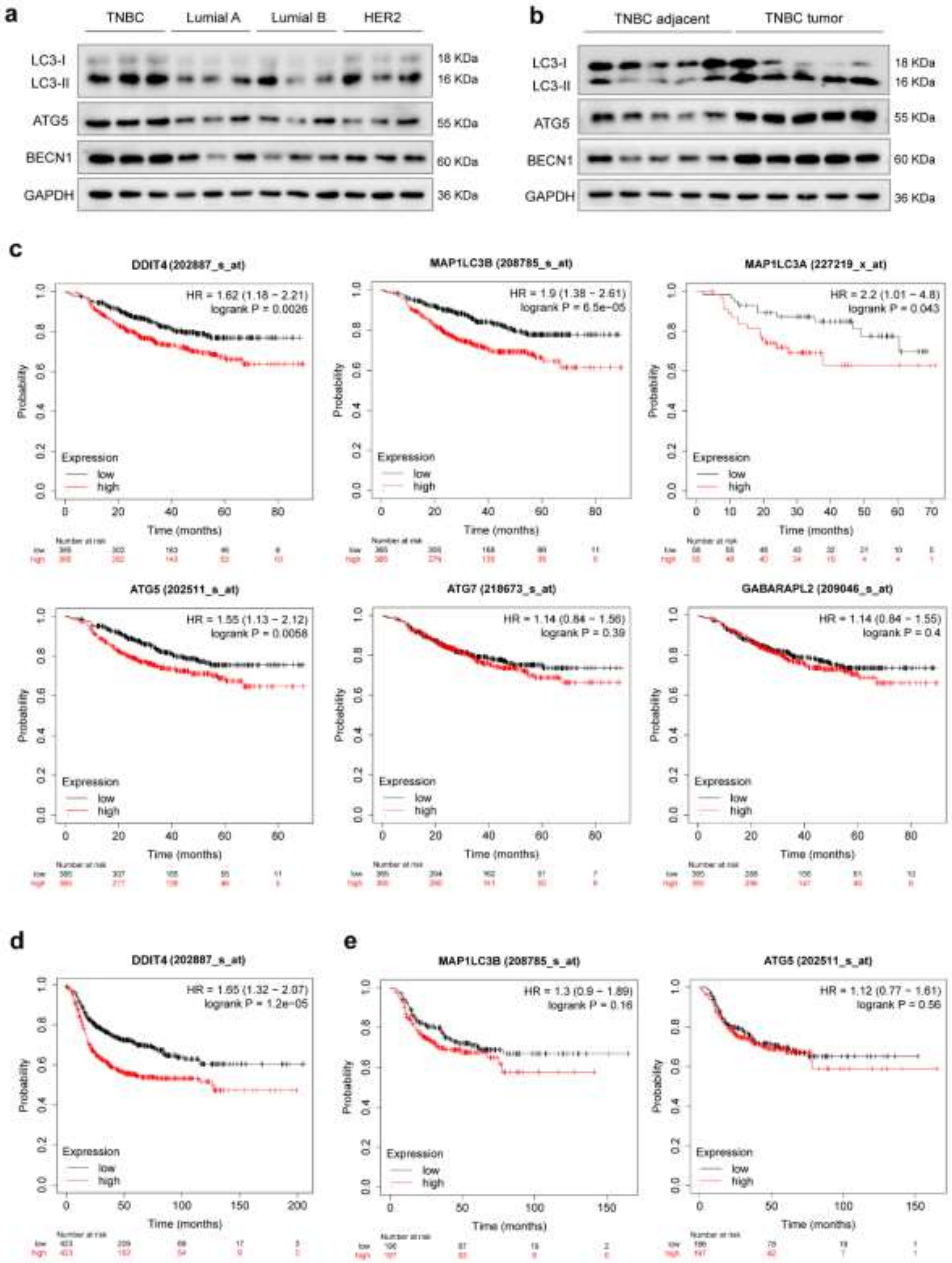

Supplementary Fig. 5 Elevated levels of DDIT4 and autophagy-related genes correlate with poor relapse-free survival in breast cancer patients. a Western blot was used to detect indicated proteins in three tumor tissues of different type of breast cancers. **b** Western blot was used to detect indicated proteins in five pairs of TNBC

tumor and adjacent tissues. **c** Kaplan–Meier analysis showed the association between expression of DDIT4 or autophagy-related genes (MAP1LC3B, MAP1LC3A, ATG5, ATG7, GABARAPL2) and relapse-free survival of breast cancer patients received neoadjuvant chemotherapy. **d** Kaplan–Meier analysis showed the association between DDIT4 expression and relapse-free survival of basal-like breast cancer patients received systemic treatment. **e** Kaplan–Meier analysis showed the association between expression of MAP1LC3B or ATG5 and relapse-free survival of basal-like breast cancer patients received chemotherapy.

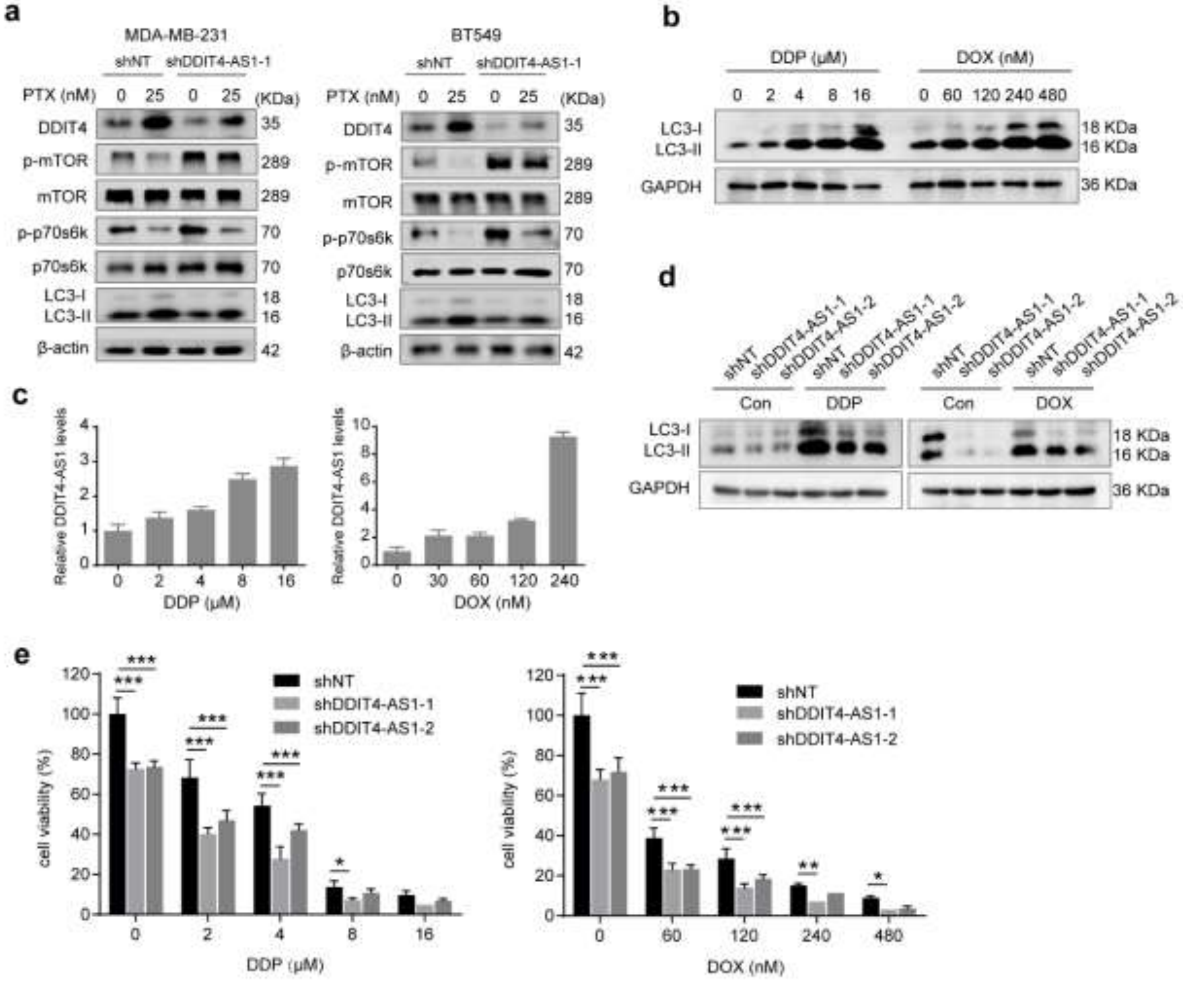

Supplementary Fig. 6 DDIT4-AS1 attenuates chemosensitivity of breast cancer cells to chemotherapeutic agents. a Western blot showing the effects of DDIT4-AS1 knockdown on indicated proteins in MDA-MB-231 and BT549 cells treated with PTX

for 48 h. **b** MDA-MB-231 were treated with cisplatin (DDP) or doxorubicin (DOX) as indicated, and then the level of LC3 and GAPDH proteins was examined by western blot. **c** MDA-MB-231 were treated with DDP or DOX as indicated, and then the level of DDIT4-AS1 was examined by qRT-PCR. β-actin was the internal control. **d** Western blot showing the effects of DDIT4-AS1 knockdown on LC3 expression in MDA-MB-231 cells treated with DDP or DOX. **e** Cell viability was analyzed by CCK-8 assay after DDP or DOX treatment for 48 h in DDIT4-AS1 knockdown cells. *$p < 0.05$, **$p < 0.01$, ***$p < 0.001$.

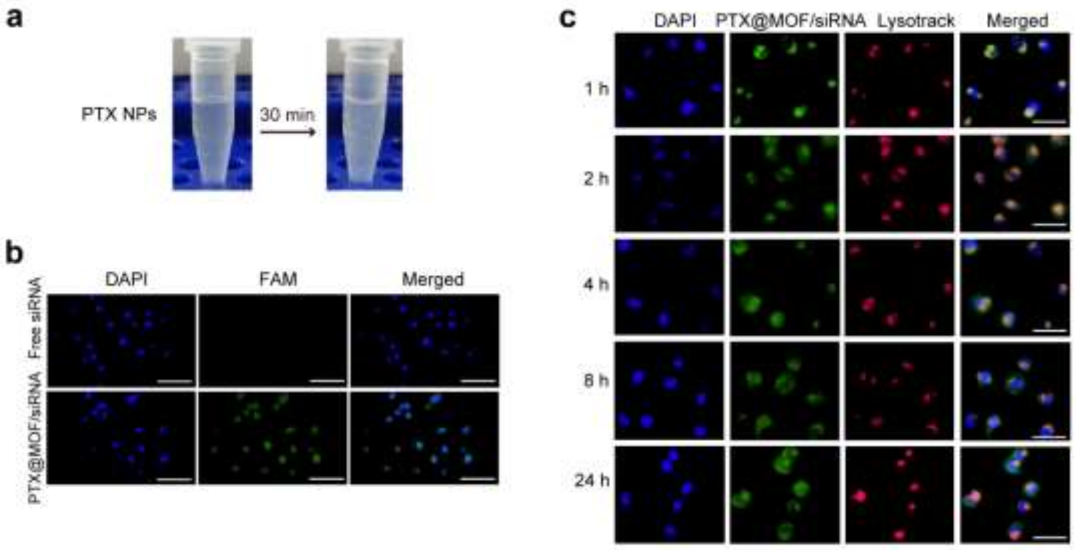

Supplementary Fig. 7 Biological behavior of PTX@MOF/siDDIT4-AS1 NPs. a The insolubility of paclitaxel in aqueous solution. **b** Fluorescent images of BT549 cells after incubating with free siDDIT4-AS1 and PTX@MOF/siDDIT4-AS1 for 4 h. Scale bar, 100 μm. **c** Costaining of endo/lysosomes to evaluate the endo/lysosomes escape in BT549 cells. Scale bar, 50 μm.

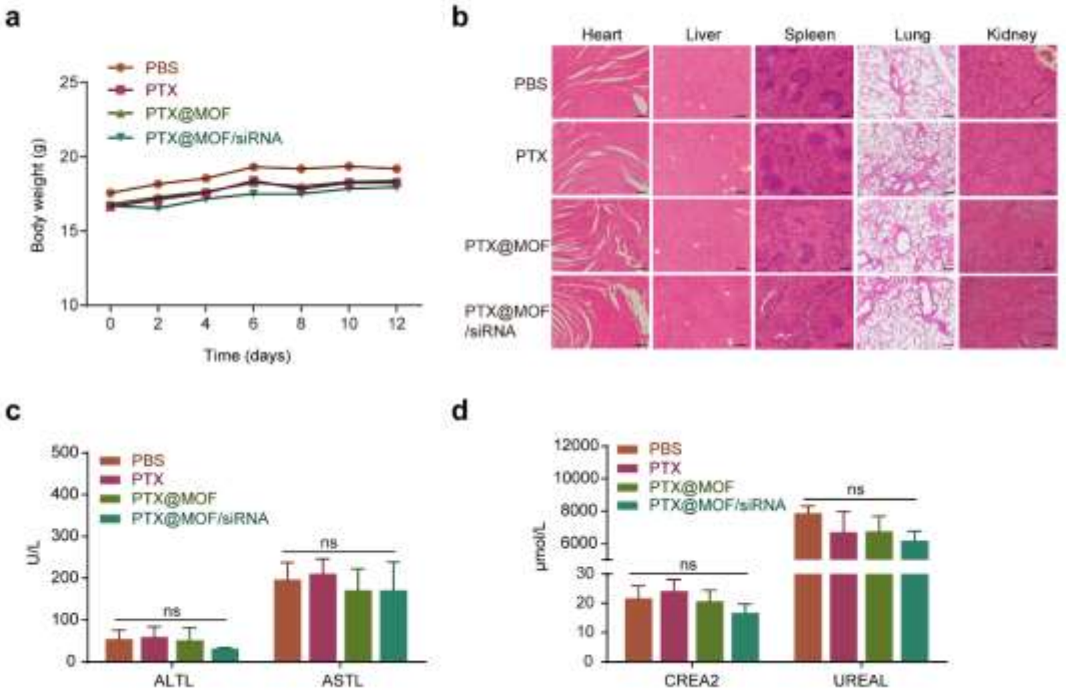

Supplementary Fig. 8 Toxicity analysis of PTX@MOF/siDDIT4-AS1 NPs. a Average body weights of mice during different treatments. **b** Histology images of H&E staining slices for the major organs (heart, liver, spleen, lung, and kidney) obtained from mice with indicated treatments. Scale bar, 500 μm. **c** Evaluation of the hepatotoxicity of various treatments by measuring the serum levels of ALT and AST. **d** Evaluation of the nephrotoxicity of various treatments by measuring the serum levels of CREA2 and UREAL.

Table S1. The selected candidate lncRNAs for subsequent verification.

Id	Gene	Con	EBSS	Fold Change	log_2 FoldChange	p-val
NR_102422	AOC3	22.18768287	198.2482171	8.935057268	3.159476976	1.26235E-06
NR_015421	LOC154761	26.07981026	158.3853481	6.07310201	2.602433602	1.96252E-05
ENST00000596379.1	LINC02560	3.83926084	157.7723659	41.09446389	5.360872147	0.000235237
ENST00000576234.1	AC127496.1	73.9199819	521.4713437	7.054538304	2.818551666	2.06176E-12
ENST00000573602.1	AC127496.1	18.726028	454.5916825	24.27592666	4.601454462	7.36842E-12
ENST00000562471.1	AL512274.1	16.67563243	137.8244487	8.265020793	3.047018449	0.001922023
ENST00000491934.2	DDIT4-AS1	360.5066471	8287.827467	22.98938878	4.522896204	1.83772E-21

Table S2. Relationship between DDIT4-AS1 expression and clinical features in breast cancer

Characteristic	Total	DDIT4-AS1 expression		χ^2	*p*
		Low (n =40)	High (n =40)		
**Gender**				0.3463	0.5562
Female	77	39	38		
Male	3	1	2		
**Age(years)**				0.8333	0.3613
≥50	32	18	14		
< 50	48	22	26		
**Tumor size**				3.6522	0.1611
≥10	26	9	17		
5<<10	42	24	18		
≤5	12	7	5		
**Histologic differentiation**				1.8291	0.1763
Well	70	37	33		
Poor	10	3	7		
**Lymph node metastasis**				3.1641	0.0753
Yes	21	6	15		
No	59	34	25		
**TNM stage**				6.3731	**0.0116 ***
I	31	21	10		
II/III	49	19	30		
**Tumor number**				0.1054	0.7454
≥2	11	5	6		
≤1	69	35	34		
**Ki67(%)**				5.9521	**0.0147***
≥30	24	7	17		

< 30	56	33	23

Statistical analyses were carried out using Pearson χ^2 test. *p<0.05 was considered significant.

Table S3. The expression information of DDIT4-AS1 and DDIT4 mRNA from the RNA sequencing data.

id	Gene	231-Con	231-EBSS	Fold Change	$\log_2$Fold Change	p val	p adj
ENST00000491934.2	ENSG00000269926.1	360.51	8287.83	22.99	4.52	1.84E-21	2.55E-18
NM_019058	DDIT4	729.52	16851.15	23.10	4.53	2.98E-21	4.02E-18

Table S4. The binding ability between DDIT4 mRNA or DDIT4-AS1 with different RNA binding proteins.

	**DDIT4 mRNA**		**DDIT4-AS1**	
Protein ID	RF Classifier	SVM Classifier	RF Classifier	SVM Classifier
**TTP(ZFP36)**	0.9	0.716	0.8	0.861
**HNRPD(AUF1)**	0.9	0.663	0.75	0.75
**KHSRP**	0.8	0.651	0.75	0.806
ELAV2(Hel-N1)	0.8	0.469	0.8	0.709
TIA1	0.8	0.457	0.85	0.795
ELAVL4(HuD)	0.75	0.52	0.75	0.773
BRF1	0.75	0.468	0.75	0.71
ELAV1(HuR)	0.75	0.412	0.75	0.683

Table S5. Target sequences of shRNAs and siRNAs.

shRNA target sequence	
shNT	TTCTCCGAACGTGTCACGT
shDDIT4-AS1-1	CAGCTCAACTCTGCAGTACAC
shDDIT4-AS1-2	AAGCCTTTGTTTCATGCTACA
siRNA target sequence	
siNC	Ribobio

siZFP36	ACCGACGATATAATTATTATA
siAUF1	CGCCTACTACTCACACTACTA
siKHSRP	GGGUCCCUCUGAAGUUUAAdTdT
lncRNA siRNA #1	CACAUCCAGCUGGAAGCCUUTT
BECN1 siRNA	CTCAGGAGAGGAGCCATTTdTdT
ATG5 siRNA	GCAACTCTGGATGGGATTG
DDIT4 siRNA	ACACTTGTGTGCCAACCTG
TCF4 siRNA #1	GCUCUGAGAUCAAAUCCGAdTdT
TCF4 siRNA #2	GCACUUGCUUCCGAUCUAUUdTdT
Smart silencer target sequence	
491934	CACATCCAGCTGGAAGCCTT<br>CCTACAACAGGTCATAACAA<br>AAGTTCAACACTCCTCAATG<br>CTACAACAGGTCATAACAA<br>CTGCCTACAACAGGTCATA<br>CAACAGGTCATAACAAAAA
573602	GGTTTGTCATGCTGACACT<br>CCTTCTCTGTCCTCACAGT<br>TGGAAGCTTCTGTGGTCTT<br>ATGCTGACACTGATGACAGA<br>ACCCTCTAGCTCATTATGTA<br>ACTCATGCCTGCAGTAACTG
576234	GCCTATCCACTGCTTTCAA<br>CTCAGCCAATCTCCTCTTT<br>CGACGACAATAAATTCTCT<br>CTTCTGCACTCTACCAGCTT<br>CTCCTATCCTCTCTCTGCCC<br>CATCACTGCTGTACACCCTG
596379	GCCTATCCACTGCTTTCAA<br>CTCAGCCAATCTCCTCTTT<br>CGACGACAATAAATTCTCT<br>CTTCTGCACTCTACCAGCTT<br>CTCCTATCCTCTCTCTGCCC<br>CATCACTGCTGTACACCCTG
